# Isoflavones: Promising Natural Agent for Cancer Prevention and Treatment

**DOI:** 10.1002/fsn3.70091

**Published:** 2025-03-11

**Authors:** Muhammad Hammad Ul Hassan, Muhammad Shahbaz, Muhammad Imran, Ushna Momal, Hammad Naeem, Ahmed Mujtaba, Muzzamal Hussain, Muhammad Junaid Anwar, Suliman A. Alsagaby, Waleed Al Abdulmonem, Tadesse Fenta Yehuala, Mohamed A. Abdelgawad, Ahmed H. El‐Ghorab, Samy Selim, Ehab M. Mostafa

**Affiliations:** ^1^ Department of Food Science and Technology Muhammad Nawaz Shareef University of Agriculture Multan Pakistan; ^2^ Department of Food Science and Technology University of Narowal Narowal Pakistan; ^3^ Post Harvest Research Centre Ayub Agricultural Research Institute Faisalabad Pakistan; ^4^ Department of Food Science and Technology, Faculty of Engineering Sciences and Technology Hamdard University Islamabad Campus Islamabad Pakistan; ^5^ Department of Food Science Government College University Faisalabad Faisalabad Pakistan; ^6^ Department of Food Science and Technology, Faculty of Food Science and Nutrition Bahauddin Zakariya University Multan Pakistan; ^7^ Department of Medical Laboratory Sciences, College of Applied Medical Sciences Majmaah University Al‐Majmaah Saudi Arabia; ^8^ Department of Pathology, College of Medicine Qassim University Buraidah Saudi Arabia; ^9^ Faculty of Chemical and Food Engineering, Bahir Dar Institute of Technology Bahir Dar University Bahir Dar City Ethiopia; ^10^ Department of Pharmaceutical Chemistry, College of Pharmacy Jouf University Sakaka Aljouf Saudi Arabia; ^11^ Department of Chemistry, College of Science Jouf University Sakaka Saudi Arabia; ^12^ Department of Clinical Laboratory Sciences, College of Applied Medical Sciences Jouf University Sakaka Saudi Arabia; ^13^ Department of Pharmacognosy, College of Pharmacy Jouf University Sakaka Saudi Arabia; ^14^ Pharmacognosy and Medicinal Plants Department, Faculty of Pharmacy (Boys) Al‐Azhar University Cairo Egypt

**Keywords:** alternative medicine, anticancer properties, apoptosis, cell proliferation, isoflavone, phytochemicals, signal transduction pathways

## Abstract

Isoflavones are currently being investigated by researchers in order to demonstrate their ability to prevent the proliferation of cancer cells. The current review aimed to demonstrate the potential of isoflavones to eliminate cancerous cells in the stomach, liver, lung, breast, and prostate, as their anticancer properties are due to the ability to block the signaling pathways of the extracellular signal‐controlled kinase (MAPK/ERK) and proteasome (PI3K/AKT/mTOR). Isoflavones can inhibit the cell division of various cancer cells. Isoflavones can block the androgen receptor (AR), a protein that is required for the growth and dissemination of prostate cancer. It initiates the caspase cascade and obstructs the production of new proteins to eliminate lung cancer cells. These inhibit colon cancer cells by entering their G2/M cell cycle phase and inducing apoptosis. These are also known to inhibit the production of cyclin‐dependent kinase 2 and cyclin B1, two proteins that are related to an enhanced risk of colon cancer. These suppress the breakdown of cyclin B1 and CDK2 to stop the development of cancer. Preclinical evidence consistently supports the efficacy of isoflavones in suppressing tumor growth; however, human clinical trials show variability due to differences in bioavailability, metabolism, and dosage. Despite their promise as alternative or adjunctive cancer therapies, limitations such as low solubility, interindividual metabolic variations, and inconsistent clinical outcomes necessitate further large‐scale, controlled trials. Future research should focus on improving bioavailability and exploring synergistic effects with conventional therapies.

## Graphical abstract

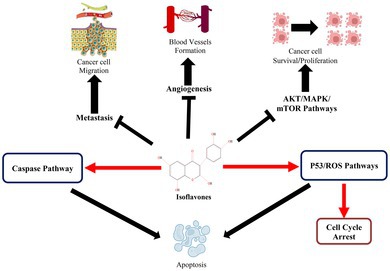

## 1 Introduction

Phytoestrogens, often called “dietary estrogens,” are naturally occurring substances that can bind to estrogen receptors and show structural similarities with mammalian estrogen (17‐β‐estradiol). The primary classes of phytoestrogens include flavonoids, isoflavonoids, stilbenoids, and lignans. Biologically active aglycones are included in isoflavones (Raheja et al. 2018). Numerous studies have demonstrated that populations of Korea and Japan consuming high levels of isoflavones (~30 mg of isoflavones per day) have a lower prevalence of metabolic syndromes, particularly obesity (Dutra et al. 2021). Isoflavones are distinguished by a structure that can exhibit nonsteroidal estrogen‐like behavior on human cells. The inclusion of isoflavones and their constituents in a variety of biological processes indicates that they have the potential to affect the expression of genes at the epigenetic level and influence multiple metabolic pathways. Smeriglio et al. (2019) have shown that isoflavones have beneficial health effects on a variety of diseases, particularly in the prevention of hormone‐related malignancies, osteoporosis, postmenopausal symptoms, and coronary heart and neurological diseases. Due to their morphological similarity to the estrogen‐like composite 17β‐estradiol, phytoestrogens are the collective term used to refer to them. Beans, alfalfa, red and white clover, and soy are among several vegetables containing isoflavones, which mainly occur in legumes (Fabaceae family). The most significant nutritional sources of isoflavones in the human diet are soy‐derived food products, soy flour, soybeans, soy granules, fermented soy, and soy beverages, including products such as tempeh and miso. Nuts, legumes, veggies, and fruits also contain isoflavones, albeit in lower amounts. Along with genistein, daidzein, and glycitein, the most common isoflavones in food are biochanin A and formononetin (FT) (Gómez‐Zorita et al. 2020).

Pulses are rich in isoflavones, the most common of which is genistein. Arrowroot and soybeans are the legumes that have the highest levels of genistein. In contrast to soybeans, genistein is present in very small amounts in other dietary sources. In estrogen receptor‐positive (ER+) cancers, such as breast cancer, the isoflavone genistein possesses tumor‐suppressing properties. The inhibition of enzyme activity, including mitogen‐activated kinase, tyrosine protein kinase, and DNA polymerase II, causes cell development to be suppressed. Moreover, among the several physiological functions of isoflavones, their antioxidant effects are particularly significant (Kim 2021). Daidzein is found in tofu, soy protein isolates, soy flour, textured soy protein, and infant formulas with soy products. Half a cup of miso has 22 mg of daidzein, three ounces of tofu has 8 mg, three ounces of tempeh has 15 mg, and one cup of soy milk has 7 mg of daidzein. Daidzein is a prospective isoflavone that has the potential to fulfill a dual function by replacing or suppressing mammalian estrogens and their associated receptors. This is because its chemical structure is similar to that of these hormones. However, daidzein also possesses additional biological functions that are not dependent on the estrogen receptor (ER), including the capacity to elicit apoptosis, modulate the immune response, and lower oxidative damage. These functions have a direct connection with its anticancer properties (Alshehri et al. 2021).

Leguminous plants, such as red clover ( *Trifolium pratense* ), are the sources of biochanin A, a bioactive component or isoflavone. Molecularly active biochanin at various phases of the cell cycle concentrates on a signal transduction route involved in the division of cells, development nuclear factor kappa B (NF‐κB), and peroxisome proliferator‐activated receptor gamma (PPAR γ). Although it increases ER development in other cellular models, biochanin A reduces the activation of mitogen‐activated protein kinases (MAPKs) and Akt in cancer cells (Raheja et al. 2018).

**FIGURE 1:**
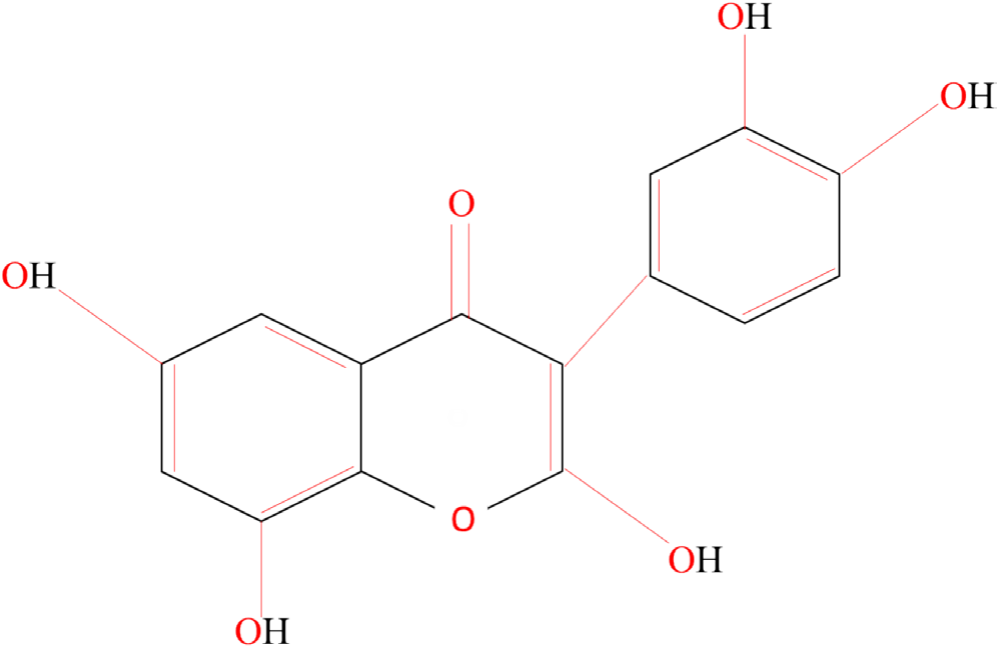
Chemical structure of isoflavones.

FT belongs to the isoflavones group and is important due to its biological properties such as antioxidant, anticancer, and anti‐inflammatory properties. Soy and red clover are the primary sources of formonetin (Dutra et al. 2021). As a natural by‐product of metabolism, free radicals are perpetually generated within our bodies. It has been demonstrated in numerous studies that isoflavones possess antioxidant properties that are equivalent to those of the prominent antioxidant vitamin E. Free radicals are neutralized and disintegrated or chelated by antioxidants. By reducing the possibility of free radical interference with DNA, isoflavones’ anti‐inflammatory properties can lower the risk of cancer. In addition, isoflavones have strong antioxidant activity in several environments, including both lipophilic and aqueous phases; this is due to various antioxidant processes. The primary method by which isoflavones benefit lipid profiles is by inhibiting lipid peroxidation, especially with low‐density lipoprotein (LDL). Multiple studies have demonstrated that lowering blood low‐density lipoprotein‐cholesterol, total cholesterol, and triglycerides is a direct result of eating soy protein. As a result, chronic disorders like cholesterol and cardiovascular disease (CVD) become more prevalent. The presence of an excess of isoflavones in serum has also been associated with legitimate concerns that it can aggravate other hormone‐related health problems. In general, the consumption of moderate quantities of soy foods that are minimally processed and traditionally prepared may provide modest health benefits while reducing the likelihood of adverse health effects (Zaheer and Humayoun Akhtar 2017). The chemical structure of isoflavone is presented in Figure 1.

The potential health benefits of isoflavones, particularly in neuroprotection and chronic disease management, are supported by different research (Kim 2021). Isoflavones, such as genistein and daidzein, exhibit various biological activities that may contribute to health improvements, particularly in postmenopausal women and individuals with metabolic disorders (Chen and Chen 2021). Isoflavones have been shown to protect against neurodegenerative diseases by modulating neurotransmitter systems and exhibiting antioxidant properties. Experimental studies indicate that isoflavones can interact with estrogen receptors, enhancing neural plasticity and reducing neuroinflammation (Gong et al. 2024). Genistin, a prominent isoflavone, has demonstrated cardioprotective effects and may reduce the risk of osteoporosis and metabolic syndrome (Islam, Ahmed, et al. 2020; Islam, Islam, et al. 2020). Dietary intake of isoflavones is linked to lower incidences of cardiovascular diseases and hormonal cancers (Popa and Rusu 2017). Isoflavones exert their effects through various mechanisms, including anti‐inflammatory and anti‐apoptotic activities, which are crucial for maintaining cellular health (Danciu et al. 2017). Human intervention studies suggest that isoflavone supplementation can positively influence gene expression related to energy metabolism and inflammation (Velpen 2014).

The current review aimed to explore the potential of isoflavones as natural anticancer agents, highlighting their mechanisms of action, biological effects, and therapeutic applications. The review examined the role of isoflavones in different cancer types, that is, stomach, liver, lung, breast, prostate, and colon cancers, by analyzing their impact on cell signaling pathways, apoptosis induction, and cell cycle regulation during in vitro, in vivo, and clinical studies. Additionally, the bioavailability and metabolic challenges of isoflavones, along with their potential integration into conventional cancer therapies, have also been assessed.

## 2 Isoflavone Metabolism and Anticancer Activity of Metabolites

The metabolism of isoflavones in the human body involves complex interactions between dietary intake, gut microbiota, and metabolic pathways (Huang et al. 2022). Isoflavones, primarily found in soy products, are metabolized into various bioactive compounds, influencing health outcomes (Garg et al. 2016). Isoflavones, such as daidzein and genistein, are metabolized by gut bacteria, particularly strains from the Eggerthellaceae family, which convert daidzein into equol, a metabolite with distinct estrogenic properties (Soukup et al. 2021). The microbial metabolism of isoflavones can lead to different metabolite profiles, classifying individuals into distinct metabotypes based on their capacity to process these compounds (Soukup et al. 2023). Epidemiological studies suggest that high isoflavone intake is linked to reduced risks of hormone‐related cancers, cardiovascular diseases, and menopausal symptoms (Sohn et al. 2021). The metabolic products of isoflavones can modulate nutrient metabolism, potentially enhancing energy expenditure and glucose tolerance (Aoi et al. 2021).

Isoflavones are initially present in foods as glycosylated forms, which are not readily bioavailable. Microbial metabolism, particularly by lactic acid bacteria (LAB), plays a crucial role in converting these forms into more bioactive aglycones, that is, daidzein and genistein, and further into metabolites, that is, equol, which have greater biological activity and potential anticancer effects (Langa et al. 2023). In human endothelial cells, genistein and daidzein are metabolized into methoxy‐genistein‐glucuronides and sulfates, which are important for their cardioprotective and anticancer effects. Equol is absorbed, not further metabolized in endothelial cells, suggesting its direct role in exerting biological effects (Toro‐Funes et al. 2015). Isoflavones exhibit anticancer properties by inhibiting cell growth, invasion, and inducing apoptosis in cancer cells, that is, melanoma (Liang et al. 2024). Their structural similarity to estrogen allows them to modulate hormone‐related pathways, potentially reducing the risk of hormone‐dependent cancers. While the metabolism of isoflavones enhances their bioavailability and biological activity, the variability in individual metabolism and the influence of gut microbiota can lead to differences in their efficacy as anticancer agents (Aboushanab et al. 2022).

### 2.1 Anticancer Perspective of Isoflavones

**FIGURE 2:**
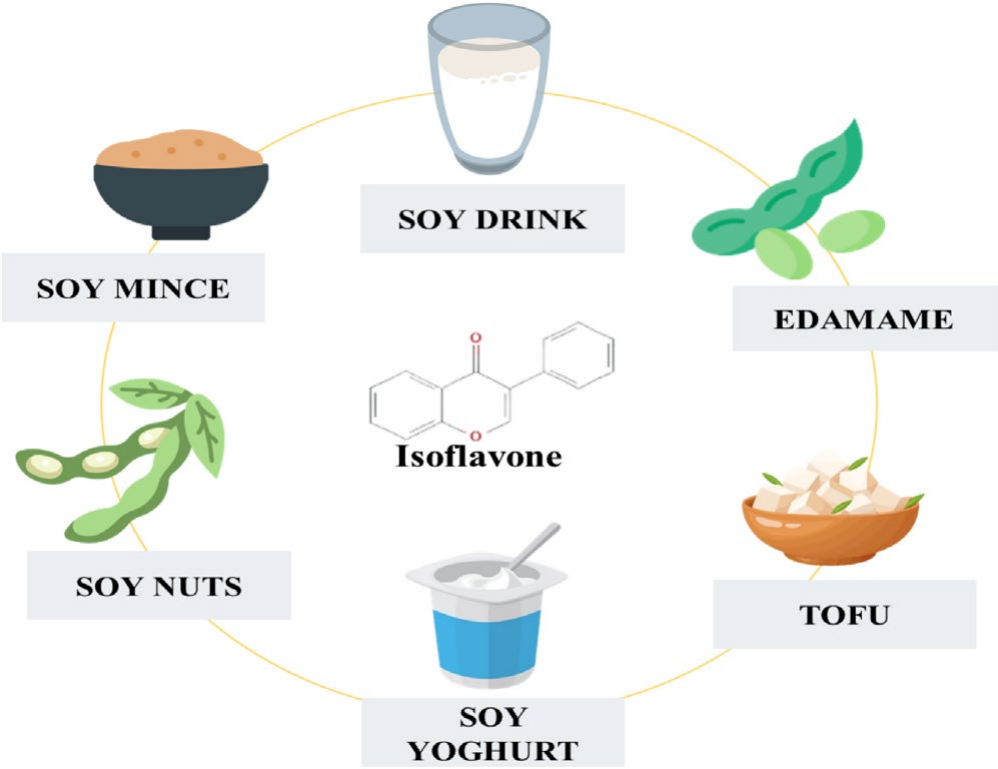
Soy and soy‐based products as natural sources of Isoflavones.

Naturally occurring flavones like isoflavone may be found in several fruits and vegetables and legumes like soybeans and chickpeas. Antioxidants help to prevent diseases caused by free radicals, which are substances lacking stability, from damaging cells and accelerating their aging. Antioxidants eliminate free radicals responsible for oxidative damage. According to a recent study, it has been shown that it may possess antioxidant qualities and aid in the elimination of harmful free radicals (Wang et al. 2016). Different natural sources of isoflavones are shown in Figure 2.

Its potential as a medicinal agent in defending against malignancies is supported by its anticancer properties. Research has demonstrated that isoflavones suppress the proliferation of breast, prostate, colon, and lung carcinomas. It achieves this by triggering apoptosis and cell cycle arrest, both of which eventually result in the death of tumor cells (Anwanwan et al. 2020). Chronic inflammation significantly affects the progression and spread of cancer. Studies have demonstrated that isoflavones amplify the susceptibility of tumor cells to chemotherapy medicines, hence increasing the effectiveness of chemotherapy. Isoflavone regulates the expression of cancer suppressor genes, that is, p53 and p21, which are crucial in the prevention of cancer development (Xu et al. 2022). Isoflavones have the potential to modify the mechanisms of progression and metastasis pathways in a way that they inhibit cancer cell survival and proliferation (Sia et al. 2017). Both the detection and treatment of cancer depend primarily on the immune system. It is suggested that an imbalance between pro‐inflammatory and anti‐inflammatory chemicals, which leads to the underlying inflammatory disorder, involves both factors. There is a suggestion that isoflavones can enhance immunological function by activating many immune system components, such as macrophages, natural killer cells (NK), and T cells (Park et al. 2019).

The damaged proteins and organelles are broken down during autophagy, which is a part of the cellular process. It stops cancer from spreading by keeping cells in a stable condition. The anticancer properties of isoflavones can be attributed to their recognized capacity to stimulate autophagy. Moreover, research has revealed that curcumin, resveratrol, and green tea catechins, together with isoflavones, have a synergistic interaction with other chemicals with anticancer effects. The combined research has the potential to diminish the harmfulness of these treatments while also improving their overall efficacy in combating cancer. Due to its low toxicity and abundant presence in many food sources, this polyphenol is in great demand for future investigation and advancement (Shafiee et al. 2016). The cell signaling pathways linked in the development and growth of cancer were linked with isoflavones' potential to suppress cancer cell growth, activate apoptosis, and regulate cell proliferation. The potential of isoflavones to enhance the effectiveness of other cancer medications has been recognized. The study investigates the antioxidant properties of isoflavones in preventing breast, prostate, and lung cancer (Alipour and Karimi‐Sales 2020).

### 2.2 Bladder Cancer

Around 430,000 cases of bladder cancer (BC) are reported each year, making it the ninth most common disease in the world. In terms of yearly deaths, it ranks as the 13th most common cancer. This cancer is more frequent in males than in females; in fact, it ranks sixth among male cancers worldwide. When it comes to male cancers, bladder cancer ranks fourth in the United States. Urothelial bladder cancer is the most prevalent subtype. Approximately, 75% of patients have a nonmuscle‐invasive disease that is restricted to the bladder mucosa/submucosa (Cumberbatch et al. 2018). In vitro and animal research have indicated that isoflavones have anti‐inflammatory and anticancer characteristics (Wada et al. 2018). In plants, the breakdown of phenylalanine leads to the production of the cinnamyl coenzyme A side chain, which in turn causes cyclization and the generation of isoflavones. Pulses, including locust horn and mountain bean root, are rich sources of the isoflavone genistein. Genistein stops bladder cancer cells from multiplying by increasing CDKN1A (p21/WAF1) and decreasing cyclin A and cyclin B1. This causes the cell cycle to stop in the G2/M stage. In addition to that, genistein boosts ROS production in T24 bladder carcinomas by impeding the PI3K/AKT signaling mechanism. By controlling apoptosis through the ATM/NF‐κB/suppressor of NF‐κB kinase (IKK) pathway, genistein indirectly makes bladder carcinomas more responsive to hydroxycamptothecin treatment both in the laboratory and in living organisms. Puerarin, an additional representative isoflavone, was extracted from *Radix puerariae*. In T24 bladder carcinomas, puerarin promotes apoptosis and inhibits proliferation. Additionally, puerarin reduces bladder cancer viability by blocking the G0/G1 cell cycle barrier, which inhibits p70S6K and mTOR phosphorylation but has no effect on the protein levels of these proteins. Moreover, puerarin increases the expression of miR‐16, a microRNA that downregulates COX‐2 expression through inactivation of the NF‐κB signaling pathway. As a result, T24 bladder carcinomas become less useful for daily consumption (Xia et al. 2021). Chen and Chen (2021) depicted that the consumption of soy isoflavones is linked with the reduction in bladder and endometrial cancer.

The researchers took prunetin to establish its efficacy on the RT‐4 cells derived from bladder cancer. The cell cultures were used in examining the cytotoxicity and nitric oxide synthase activity of prunetin. RT‐PCR techniques, which are real‐time polymerase chain reaction techniques for the apoptotic genes, were employed to establish whether they worked. A flow cytometer was used for cell cycle experiments to obtain the intracellular apoptotic rate. The results show that RT‐4 cells are cytotoxic to prunetin with the IC50 value 21.11 μg/mL. Prunetin produced death and prevented the cell cycle at the G0/G1 phase. Treatment with prunetin at the doses of 21.11 and 42.22 μg/mL, respectively, appears to increase the expression of genes TNF‐α in RT‐4 and CASP3, respectively. In macrophage‐driven inflammatory conditions, nitric oxide was significantly decreased at an IC50 of 5.18 μg/mL (Köksal Karayildirim et al. 2021). The in vitro antitumor properties of the soy isoflavone genistein against human bladder carcinoma T24 cells resulted in genistein‐stimulating apoptosis and also arresting cell cycles at the G2/M phase. Genistein decreased levels of cyclin A and cyclin B1, but it raised intensities of p21WAF1/CIP1, which is an inhibitor of the cyclin‐dependent kinase (Cdk) that is linked to Cdk2 and Cdc2. In addition, genistein facilitated the fragmentation of poly (ADP‐ribose) polymerase and stimulation of caspases (caspase‐3, ‐8, and ‐9). Nevertheless, the pan‐caspase inhibitor effectively reduced the genistein‐induced apoptosis, underscoring the essential role of caspase in facilitating genistein's capacity to trigger cell death. Furthermore, genistein enhanced cytosolic cytochrome c release by reducing mitochondrial integrity and raising the Bax/Bcl‐2 ratio. LY294002, a phosphoinositide 3‐kinase (PI3K)/Akt inhibitor, increased the apoptosis‐causing actions of genistein. In contrast, genistein directly suppressed the PI3K/Akt signaling pathway. N‐acetyl cysteine (NAC) effectively decreased ROS production to a greater extent than genistein. Additionally, NAC reversed the effects of genistein on cell death, cell cycle arrest at the G2/M phase, and PI3K/Akt signaling. The findings demonstrated that genistein‐induced apoptosis in T24 cells derived from human bladder cancer. The effect was associated with the inhibition of G2/M phase transition in the cell cycle caused by ROS‐dependent PI3K/Akt signaling pathway regulation (Park et al. 2019).

Another study also evaluated the molecular mechanism and potential anticancer benefits of FT on BCa cells. Cell proliferation was evaluated by multiplex TT, Hoechst 33258 stain, and western blotting; cell apoptosis and invasiveness by western blotting; and miR‐21 effect by western blotting. Protein expression levels of p‐Akt and phosphatase, PTEN, were measured. The results presented that FT inhibits the growth of T24 cells both in time‐ and dose‐dependent manners. The morphological change was obvious in the T24 cells after exposure, and the invasiveness was reduced. FT also significantly inhibited the function of miR‐21 in the T24 cells by increasing PTEN and decreasing p‐Akt. These findings suggest that FT inhibits BCa carcinogenesis in vitro, possibly via miR‐21 modulating the PTEN/Akt pathway (Wu et al. 2017). Researchers have used the in vitro cell line and in vivo xenograft animal model to determine the molecular pathways and effects of daidzein on bladder carcinoma cells. Both the concentration and the exposure time of daidzein were determinants for cell viability. Daidzein induced death in human bladder cancer cells through G1/S cell cycle arrest, leading to a notable reduction in colony‐forming ability. Studies also confirmed that daidzein effectively suppressed the propagation of RT112 cell xenograft cancers in nude mice. The efficacy of daidzein was attributed to the significant downregulation of the FGFR3 signaling mechanism, as indicated by mechanism studies. In conjunction with the reduction of certain apoptosis‐suppressing molecules, the phosphorylation levels of Akt, FGFR3, and Erk proteins were inhibited during daidzein treatment. The functioning of daidzein in contrast to bladder tumor was impaired by the knockdown of endogenous FGFR3, indicating that the expression of daidzein was primarily modulated by the FGFR3 pathway. Furthermore, the activity model of daidzein was comparable to that of the FGFR3 antagonist PD173074 in RT112 cells. In summary, the findings indicated that daidzein has the potential to impede the growth of bladder cancer and may act as a new operative chemotherapeutic agent for the management of bladder carcinoma (He et al. 2016).

### 2.3 Blood Cancer

The bone marrow is the site of the initiation of the majority of blood cancers, which are also referred to as hematologic malignancies. Blood cells are generated and disseminated through the bloodstream. Leukemia is the most prevalent form of blood malignancy that originates in blood‐forming tissues. This cancer is the uncontrolled development of blood cells, especially white blood cells present in bone marrow and, later, in the bloodstream outside of the tumor. All four forms of leukemia: acute lymphocytic (ALL), acute myeloid (AML), chronic myeloid (CML), and chronic lymphocytic are the most common forms of the disease. Regrettably, in 2012, over 350,000 new cases of leukemia were identified. The highest leukemia occurrence rates were observed in Australia and New Zealand; however, the reduced rates were detected in Western Africa for both genders. The rates were primarily higher in males than in females, with a global ratio of 1.4 of male to female. Acute lymphoblastic leukemia was the most prevalent subtype in both sexes in children and adults. Leukemia therapy is lethal and has the potential to cause damage or disrupt the normal functions of numerous organ systems, even though conventional treatments are generally effective, particularly for children (Saraei et al. 2019). Isoflavones are closely related to flavonoids, a group of secondary metabolites that possess phytoestrogen properties. These compounds are derived from plants and possess estrogenic properties. Genistein, daidzein, and phenoxodiol are among the most well‐known isoflavones, and they have been shown to have high specificity against a variety of human malignancies (Jeyaraju et al. 2016). Malignant multiple myeloma (MM) is the most prevalent bone marrow cancer in the United States. Xie et al. (2016) have reported that genistein, which is mainly from Leguminosae, blocks cellular growth, stimulates programmed cell death, and improves the inhibition of the growth of blood vessels. The bioflavonoid phytochemical constituent of red clove, biochanin A, is found to possess antimyeloma properties. Biochanin A (BCA) was discovered to have an anticancer effect against types of tumors, such as lung tumors, prostate, pancreatic, breast, osteosarcoma, and malignant melanoma (MM) (Jaina et al. 2022).

Another study was done to assess the anticancer activities of BCA, an isoflavone, in the context of multiple myeloma. It was observed that MM cells undergo significant apoptosis, and levels of cytokine expression decreased in MM cells upon treatment with biochanin A. Biochanin A also significantly decreased the population of CD38 and the expression of cancer stem cell markers, such as CD117 and CD138, in a dose‐dependent manner. Although the results showed consistent reductions in stem cell markers and invasive potentials of cells with BCA treatment, it has been visibly shown that BCA treatment can drastically reduce the U266‐induced tumor growth in the NOD/SCID mice model (Jaina et al. 2022). A mechanistic study revealed that modulation of the MAPK and NF‐κB signaling mechanisms attributes to the anticancer activities of BCA. The present investigation has potential strategies to induct BCA as a novel agent for multiple myeloma therapy with lessened toxicity and superior efficacy.

The antileukemia activity of genistein on HL‐60 cells was studied by researchers under in vitro conditions. The amount of ΔΨm, the ratio of viable cells, apoptotic cell death, ROS generation, cell cycle circulation, and calcium production was measured by flow cytometric assays. The Western blotting technique was used for the detection of proteins associated with stress on ER and cell death. Expression of GRP78, GADD153, and Calpain 1 was determined under confocal laser microscopy. The findings of the study revealed that genistein can give rise to alterations in cellular morphology, DNA wreckage, and fragmentation (cell programmed apoptosis), arrest at G2/M level, with an overall decrease in viable cells in HL‐60 cells. Genistein potentiated the production of Ca2+ and ROS with a reduced level of ΔΨm in the HL‐60 cells. This led to an elevated level of proteins that were involved in causing endoplasmic reticulum stress in the HL‐60 cells. These proteins include GADD153, Calpain 1, IRE‐1α, GRP78, caspase‐4, caspase‐7, and ATF‐6α. Furthermore, genistein also upregulated protein expression related to cellular death, including cleavage of PARP, Bax, caspase 3, and 9. Moreover, the influence of antiapoptotic proteins like Bcl‐2 and Bid was diminished. HL‐60 cells exhibited elevated levels of Calpain 1, GRP78, and GADD153 following treatment with 40 μM of genistein. During the xenografted animal model, the mice were administered genistein intraperitoneally at dosages of 0, 0.2, and 0.4 mg/kg for a duration of 28 days. Body weight of mice and tumor volume were then examined. Genistein had no impact on body weights; however, the group administered 0.4 mg/kg of genistein had a noteworthy decrease in tumor weight. Moreover, genistein enhanced the tumor‐specific expression of GRP78, ATF‐6α, Bax, Bak, and Bad. In vivo, genistein inhibited tumor characteristics and lowered cell population in HL‐60 cells by arresting the G2/M phase and inducing cell death through pathways dependent on endoplasmic reticulum stress and mitochondria (Hsiao et al. 2019).

FT is a plant isoflavone purified from *Trifolium pratense* , *Astragalus membranaceus*, *Pueraria lobate*, and *Glycyrrhiza glabra* . Another recent study examined whether the anticancer activity of FT is mediated through STAT3 and STAT5 signaling pathways using a human myeloma xenograft model of athymic nu/nu mice. According to the in vitro study, FT could induce cell death and significantly inhibit cell viability. In addition to the inhibition of downstream kinases of MM cells, including JAK2, JAK1, and c‐Src, FT inhibited STAT3 and STAT5 activation. A shift in the ratio of reduced glutathione GSH toward that of its oxidized form, GSSG, has been shown to regulate this activity and increase the formation of ROS. FT inhibited the DNA binding and nuclear migration of STAT5 and STAT3. FT not only suppressed the effect of STAT3‐mediated angiogenic, antiapoptotic, and antiproliferative genes, but also impeded cell cycle progression and interacted with the activation of caspase‐3 and PARP cleavage. FT administered intraperitoneally showed significant efficacy in suppressing cancer growth in a mouse model of multiple myeloma xenograft, deprived of any notable adverse impacts. The results concluded that FT has potent antitumor properties in MM. This impact is due to the inhibition of STAT5 and STAT3 signaling pathways, which is mediated by reactive oxygen species (Kim et al. 2018).

### 2.4 Bone Cancer

Bone cancer may appear in two different ways: primary and secondary. The kind of tumor that develops in the bones is primary bone cancer. The term “metastasized secondary bone tumor” describes a specific kind of cancer that has spread from another organ or tissue to the bones. Mesenchymal tissue, which includes spindle‐shaped stromal cells with the ability to produce bone‐like tissues, is the site of development for osteosarcoma (OS), a malignant tumor. Twenty percent of the world's initial bone malignancies are caused by it. Adolescents are really more likely to develop this type of primary malignant bone tumor. Although osteosarcoma is rare in the sacrum, spine, and pelvis, it is common in the metaphysis of long tubular bones including proximal tibia, distal femur, and proximal humerus. When osteosarcoma is first diagnosed, most individuals only have one wound. It should be noted that around 10%–20% of cancer patients already have detectable metastatic illness before the disease initially. With 85% of cases occurring in the lungs, the next most prevalent place is the bones with 8%–10% of cases and, sometimes, the lymph nodes. It is suggested that subclinical or micro metastases affect the remaining 80%–90% of cancer patients (Zhao et al. 2021). Plant‐derived bioactive compounds are a collection of active substances that exhibit a number of pharmacological functions in humans, including antioxidant, antistress, anticancer and antiviral characteristics (Zheng et al. 2024). In recent decades, isoflavones have been the focus of extensive research because of their potential beneficial impacts on numerous human diseases including cancers. They are a significant group of phytoestrogens and a class of flavonoid phenolic components. Red clover and other leguminous plants are good sources of the naturally occurring isoflavone biochanin A. It suppresses tumor growth in a wide variety of cancers, including those of the bladder, prostate, hepatoma, breast, bone, and pancreas. Biochanin A increases the apoptotic and antiproliferative properties of sorafenib in hepatocellular cancer cells. Cytochrome C interacts with apoptotic protease‐activating factor‐1 (apaf‐1) to produce an apoptosome and depends on procaspase‐9. Cell death is triggered by caspase‐9, which uses effector caspases such as caspase‐3. Caspase‐3 breaks both internal and extrinsic pathways of poly (ADP‐ribose) polymerase (PARP) upon cell death (Hsu et al. 2018).

Scientists investigated the inhibitory impact of biochanin A on the growth and division of osteosarcoma cells. Results show that biochanin A inhibits cell multiplication and colony formation in a dosage‐dependent manner while causing minimal harm to healthy cells. The concurrent use of biochanin A and doxorubicin may result in a reduction in the proliferation of osteosarcoma cells. Indicators of the destructive activity of biochanin A are apoptotic bodies, phosphatidylserine accumulation in cells at the sub‐G1 stage, caspase 3 stimulation, and PARP breakdown. Apoptosis is associated with the loss of the mitochondrial membrane potential, increased expression of Bax, release of cytochrome *c*, activation of caspase 9, and reduced expression of Bcl‐2 and Bcl‐XL. Pretreating cells with caspase‐9 inhibitors (Z‐LEHD‐FMK) partly decreased apoptosis, indicating the involvement of the fundamental mitochondrial apoptotic cascade. Nevertheless, the administration of the JNK suppressor SP600125, p38 MAPK suppressor SB203580, MEK inhibitor PD‐98059, or antioxidants vitamin E, *N*‐acetylcysteine, and glutathione could not mitigate the cell death caused by biochanin A. These findings reveal that biochanin A enhances the Bax:Bcl‐2/Bcl‐XL ratio and stimulates the crucial mitochondrial route and caspase‐9 and ‐3 in osteosarcoma cells, leading to the downregulation of cell proliferation and stimulation for death (Hsu et al. 2018).

The actions of biochanin A on the U2OS and MG63 osteosarcoma cell lines included the alteration of cell proliferation, apoptosis, invasion, and translocation. Independent experiments were performed for molecular pathway analysis, which is necessary to produce the above results. Inhibition in cell viability was shown for U2OS and MG63 cells; the inhibition was dependent on the dose and duration of exposure. The same conditions caused an increase in the G0/G1 phase cell ratio and decreased the G2/M phase cell ratio. Migration and invasion capacities were significantly downregulated, while apoptosis rates were significantly enhanced in the biochanin A‐treated MG63 and O2OS cells. Translocation, invasion, cell death, and the expressions of U2OS/MG63 cells were altered. The findings indicate that biochanin A was observed to suppress osteosarcoma with cell death regulation, proliferation, invasion, and migration (Zhao et al. 2018).

Anti‐OGS effects exerted by FN were investigated through a range of biochemical experiments and immunoassays using the human OGS cellular line, U2OS, and naked mice harboring cancer. Human data showed an increase in p‐PI3KCATyr 317, ERα, and p‐AKT Ser473 proteins in OGS test samples, and then a remarkable increase in miR‐375 content compared to OGS‐free samples. In addition, FN treatment resulted in a reduced endogenous miR‐375 level, elevated lactic dehydrogenase formation, and decreased cell proliferation in U2OS cells. Furthermore, the number of cells that evaluated positive for p‐PI3KCA Tyr317, Ki‐67, and p‐AKT Ser473 decreased after FN treatments, but the number of cells that evaluated positive for intracellular Apaf‐1 and Bax rose in a dose‐dependent way. It was beneficial to observe a decrease in cancer mass and intercellular miR‐375 expression in mice with cancer that had FN‐mediated treatment. Meanwhile, FN‐modulated animals showed enhanced Bax, Apaf‐1, and Caspase‐3 immuno‐labeled cells and proteins that were dose‐dependent. In addition, ERα, p‐AKT Ser473, and p‐PI3KCA Tyr317‐positive cells and proteins were downregulated. Combined, these findings demonstrate that FN provides satisfying benefits against osteosarcoma. The pharmacological pathway may be associated with the proliferation of apoptosis by inhibiting the intracellular miR‐375/ERα‐PI3K/AKT cascaded mechanism (Hu et al. 2019).

### 2.5 Brain Cancer

Brain tumor arises in the spinal cord or brain. Brain cancer manifests in a variety of symptoms, including speech changes, mood swings, frequent migraines, and coordination issues. Brain tumors are a type of malignancy which are limited to the central nervous system or the brain. Brain malignancies are classified into a variety of categories based on their origin, nature, growth rate, and proliferation stage. Brain tumors may be classified as malignant or benign. Pituitary cancers, meningiomas, and astrocytomas are examples of benign brain tumors that have specialized boundaries, a sluggish proliferation rate, and sometimes attack neighboring healthy cells. Neighboring cells in the spinal cord or brain are readily attacked by malignant brain cancer cells, such as oligodendrogliomas and high‐grade astrocytomas. Primary brain tumors and secondary brain tumors are the two types of brain cancer. A primary tumor starts in the brain itself. Alternatively, metastasis, also known as a secondary brain tumor, is when cancer spreads from another organ, like the lungs or stomach, to the brain (Tandel et al. 2019).

Glioblastoma is a primary brain malignancy that may occur in any part of the central nervous system (CNS), although it occurs most commonly in the temporal or frontal lobes. It differs from secondary brain cancers that originate through metastases from primary sites elsewhere—like the lungs, breast, or skin—by arising within the CNS itself (McKinnon et al. 2021). In several animal models and some clinical studies, a diet high in isoflavonoids, especially biochanin A and genistein, has been found to inhibit the development and spread of cancer in both in vitro and in vivo environments. It limits the cytotoxicity in healthy tissue, and BCA limits rat and human glioma cells effectively (Sehm et al. 2014).

The effects of BCA on energy metabolism and cancer prevention have been researched in the study. It has been depicted by the team that BCA‐VERTEX inhibited the growth of U251 cells through the mitochondria‐mediated intrinsic pathway of apoptosis. Perhaps supportive oxidant stress against GBM corresponds to increased ROS generation, decreased metabolic activity, and lowered potential of the mitochondrial membrane upon treatment with BCA. Further evaluation showed that being an inhibitor of aerobic glycolysis, BCA blocked the AKT/mTOR pathway. It inhibits metabolic reprogramming, as reported by studies, which in turn controls the growth of GBM using therapy BCA (Dong et al. 2021).

Research indicates that genistein can inhibit the proliferation of glioblastoma multiforme and medulloblastoma cells with many radio responses and TP53 mutations. It is accomplished by stopping cells in the G2/M phase of the cell cycle. The damage to DNA had no role in the cell cycle arrest that remained for at least 10 days after the medication was removed. Genistein therapy, as shown by Annexin V staining, indicated that there were no populations of necrotic or apoptotic cells. The findings showed that genistein causes a little amount of DNA damage but no cell death due to the cell cycle slowdown. According to investigations of gene and protein expression, the treated cell types show similar alterations in the experimental pathways. Genistein also blocked telomerase activity, which resulted in shortened telomeres. Genistein suppresses TR‐ and TERT‐mRNA, which in turn promotes growth arrest in brain tumor cells. This finding was the first to demonstrate that genistein interacts with telomerase inhibition (Khaw et al. 2012).

In order to enhance the effectiveness of U87MG human glioblastoma cells in fighting tumors, a separate study investigated the development of Gen‐TMZ‐NPs. These were nanoparticle systems made of poly (lactic‐co‐glycolic‐acid) and included both temozolomide (TMZ) and genistein (Gen). The physicochemical properties of the nanoparticles were analyzed using FT‐IR spectroscopy, zeta potential, polydispersity index, and mean particle size measurements. The nanoparticles consisted of Gen‐TMZ‐NPs, Gen single‐drug‐loaded PLGA‐NPs, and TMZ. Researchers investigated in a controlled laboratory environment if Gen‐NP, Gen‐TMZ‐NPs, and TMZ‐NP killed U87MG cells. IC50 results of 35.2 μg/mL in 24 h and 8.7 μg/mL in 48 h clearly show that the cytotoxicity of U87MG cells treated with the Gen‐TMZ‐NP significantly increased. Following 48 h of therapy, the Gen‐TMZ‐NP treated group exhibited a wound closure rate of 6.80%. Additionally, the BrdU incorporation test revealed a decrease in the cell proliferation and migratory activity of U87MG cells. Quantitative real‐time PCR and western blotting confirmed that Gen‐TMZ‐NP treatment caused activation of apoptotic pathways in U87MG cells. The findings indicated that Gen‐TMZ‐NPs revealed superior anticancer properties in U87MG cells, implying that the utilization of two nano drug delivery methods might be an efficacious approach for glioblastoma therapy (Yazdani et al. 2016).

### 2.6 Breast Cancer

Breast cancer is a terrible syndrome that impacts people all over the world. Statistics demonstrate that every year, there are over 14,000 new instances of breast cancer and about 8000 deaths related to this disease. The isoflavones have the potential to reduce breast cancer in pre‐ and post‐menopausal females (Boutas et al. 2022). Nutrients that are high in sugar, alcohol, and caloric content are among the numerous factors that are connected with an elevated threat of breast cancer. These isoflavones elicit free radical scavenging, modify hormonal and enzymatic activities, and produce DNA damage in breast cancer cells via regulating numerous nuclear and cellular signaling cascades. Traditional Asian foods that are rich in isoflavones include miso, tempeh, tofu, and soy milk. On the other hand, some Western diets include meats that have been fortified with isoflavones by adding soy proteins and animal derivatives. Cultured with breast cancer cells, dietary isoflavones—natural phytoestrogens display—estrogenic and antiestrogenic effects. Genistein, daidzein, and glycitein are the most well‐known soy isoflavones in the battle against breast cancer. Another mechanism by which these isoflavones could moderate breast cancer is through the inhibition of matrix metalloproteinase‐9 expression, an enzyme involved in the process of regulating breast cancer (Iqbal et al. 2018).

The dose–response meta‐analysis of the China Kadoorie Biobank (CKB) study included 30–70‐year‐old, 300,000 females from 10 different areas of China from 2004 to 2016 to evaluate breast cancer. The mean consumption of soy was 9.4 ± 5.4 mg/day among the participants. During the 10‐year study, breast cancer developed in 2289 women. The meta‐analysis depicted that an increase in 10 mg/day consumption of isoflavones reduced the 3% risk of breast cancer. The study concluded that moderate amounts of soy consumption have no linkage with breast cancer. However, the highest concentrations of soy consumption can significantly prevent breast cancer (Wei et al. 2020). Another meta‐analysis study depicted that higher consumption of isoflavones, flavones, and flavonols has the potential to reduce cancers specific to women (endometrial, ovarian, and breast; Liu et al. 2022). Asians normally have a diet that is basically composed of soy products and consume an amount of 20–30 g of soy protein daily, which translates to an intake of about 100 mg of isoflavones. In contrast, diets less traditional for Asian intake contain less than 1 g of soy protein per day. The study has examined the diverse responses of breast cancer cells to estrogen in terms of cell cycle arrest, angiogenesis, proliferation, apoptosis, epigenetic regulation, metastasis, estrogen metabolism, estrogen biosynthesis, and multi‐drug resistance. In vitro chemical structure, phytochemical metabolism, and dietary sources are also explored for their interrelationship with the signaling pathways and estrogen receptors (Basu and Maier 2018). A meta‐analysis study depicted that a 5 g/day intake of soy protein reduced the 12% risk of breast cancer (Nachvak et al. 2019).

Another study was undertaken in order to examine the anticancer effect of equol on cell lines of breast cancer and determine whether equol may show a potential role as a therapeutic agent in the treatment of this disease. The MTS test was utilized in assessing the anticancer effect of equol on the human breast cancer cell line, while the combination index was used in order to analyze this integrated effect with the current hormonal or chemotherapeutic drugs. The researchers associated the results with the anticancer impact after conducting Western blot analysis to evaluate the expression of essential proteins. According to the findings, equol had antitumor and protumor actions in two distinct phases. Low amounts promoted tumor development in hormone receptor‐positive cell lines; however, anticancer effects were typically shown after administering an incredibly high dosage to blood and tissue. When taken with tamoxifen, equol may have an antagonistic impact; however, the strength of the effect depends on the concentration of equol and the kind of cancer cells. Research has shown that equol has two effects: one that promotes tumor development and one that inhibits tumor growth. According to the findings, depending on the concentration, equol may have an antagonistic impact on tamoxifen. In contrast, equol showed no signs of antagonistic effects on other medicinal medicines (Hatono et al. 2021). Additionally, researchers studied whether the biotransformation of soybean extract (BSE) by the fungus *Aspergillus awamori* improved its antitumor effects in a different study. The BSE showed enhanced antiproliferative and antiaromatase action in breast cancer cells that overexpressed ER+ aromatase. The propagation of the cancer cells was lowered by D and G, despite being classified as mild aromatase inhibitors. Genistein was the isoflavone responsible for the symptoms caused by BSE. The biotransformation showed that soybean extract has enhanced antitumoral and antiaromatase activities in breast cancer cells. Furthermore, it expanded on the potential of soya in preventing and/or treating ER+ breast cancer (Amaral et al. 2017).

The antiproliferative ability of 12 soybean anticarcinogens was investigated by treating MDA‐MB‐231 and MCF‐7 human breast cancerous cells either individually or in combination. The findings demonstrated that when tested against MCF‐7 cells, genistin, daidzein, glycitein, and daidzin had the highest efficacy as antiproliferative drugs. Genistein and daidzin form a combination index at 50% inhibition (CI50) in MCF‐7 cells, suggesting a synergistic effect. Genistein, β‐sitosterol, and glycitein were found to contain a more potent antiproliferative effect on MDA‐MB‐231 cells. A synergistic impact was seen when genistein and β‐sitosterol were combined. This combination regimen improved the suppressive result of these bioactive anticarcinogens, potentially impeding the attack and movement of the breast cancer cells. The key mechanism involved in the anticancer response seems to be the regulation of the PI3K/Akt/mTOR pathway (Zhu et al. 2018).

In a mouse model of 4T1 breast cancer, the researchers investigated the usage of the soy isoflavone extract either alone or together with docetaxel‐affected death, treatment resistance, revascularization, and tumor volume. Random assignments of 60 female BALB/c mice were made to one of four groups, including control, soy isoflavone extract (Iso, 100 mg/kg food, 0.01%), intravenous docetaxel (10 mg/kg), or a combination of both treatments. Eight mice from each group received treatment to eliminate their breast tumors 1 week after the third injection. It covered Pgp gene and NF‐κBp65 gene expression as well as the development of vascular endothelial growth factor receptor‐2. The duration of observation was lengthened until the final seven mice passed away. Their variations in survival rates were examined. The NF‐κBp65 protein and gene expression in the Docetaxel + Iso‐treated group was noticeably lower than in the group treated with Docetaxel. Compared to the group receiving treatment with only Docetaxel, there were increased inhibitions of VEGFR2 protein expression levels in both groups receiving treatment with Docetaxel + Iso and Iso only (Hejazi et al. 2017).

### 2.7 Cervical Cancer

Cervical cancer ranks as the second most prevalent malignant tumor in women globally, presenting a substantial threat to women's health. The primary etiology of cervical cancer has been determined to be chronic infection with high‐risk human papillomavirus (HPV). Cervical cancer ranked fourth in terms of frequency and mortality among women globally in 2018 (Zhang et al. 2020). It was responsible for around 570,000 cases and 311,000 deaths. Plant‐derived natural products have been identified as the most promising oncology therapies in recent years. They have the ability to selectively eliminate tumor cells while causing minimal toxicity to normal cells. Flavonoids, which are further subtypes, include isoflavones, which have the capacity to lower tumor cell proliferation, elicit apoptosis, decrease telomerase activity, and suppress angiogenesis (He et al. 2021). Genistein and daidzein are the primary isoflavones found in vegetables, with soybeans being the most common source. Genistein has the potential to enhance the efficiency of radiation therapy for cervical cancer. Genistein has been shown to inhibit the following enzymes: (1) topoisomerase I and II, (2) tyrosine protein kinases, (3) protein histidine kinase, and (4) 5α‐reductase. It has a heterocyclic diphenolic structure. Furthermore, it causes G2M cell cycle arrest in certain cells. The cell cycle can be disrupted by genistein, which might perhaps explain the increased cell apoptosis caused by radiation. The study revealed that genistein has a radiosensitizing impact on Me180 cells and CaSki cells, along with a dosage‐dependent inhibitory impact on cervical cell lines. According to the findings, daidzein had no impact on the radiosensitivity of the most prevalent isoflavones, nor did it alter the in vitro therapy of cervical cancer. This chemical effectively hindered the propagation of HeLa cells at a concentration range of 6.25–100 μmol/L. Daidzein had several effects on human cervical cancer cells that are not hormone‐dependent, including modulation of in vitro cell cycle, telomerase activity, and cell proliferation (Moga et al. 2016).

Researchers conducted in vitro investigations to examine the anticancer properties of daidzin in contrast to human cervical carcinoma. HeLa cell lines, derived from cervical cancer, were purchased from ATCC. It was maintained in DMEM media. The MTT test was used to measure the cytotoxic impact of daidzin on the HeLa cell line. Cells were treated with a dose of 20 μM of daidzin for further study as the IC‐50 value had been established at 20 μM. Evaluation of ROS formation was conducted using DCFH‐DA staining, whereas the investigation of apoptosis induction was done using Rhodamine‐123 labeling. The vitality and apoptosis of cells were assessed using ethidium bromide and acridine orange staining. Moreover, checking by the matrigel cell adhesion test, the antiadherence of cancer cells was seen as a repressing action of daidzin. The apoptotic effects of daidzin were viewed through measurement at the levels of Caspase 8 and 9 by the ELISA method. The efficacy of daidzin in inhibiting the growth of human cervical carcinoma HeLa cells was evaluated by quantifying the proliferation of cells and inflammatory signaling proteins using quantitative polymerase chain reaction. Daidzin generated ROS and significantly affected the mitochondrial membrane permeability in the HeLa cell line. According to the matrigel cell adhesion method, daidzin‐induced apoptosis in HeLa cell lines and subsequently inhibited their adhesion potential, as indicated by the AO/EtBr staining results. Besides this, the levels of caspases 8 and 9, acting as key regulators of apoptosis, were enhanced by the administration. Daidzin quelled the inflammatory gene expression and the signaling molecule of cell proliferation. Results showed that the component daidzin had significantly reduced induced apoptosis and inflammation of HeLa cell lines of human cervical carcinoma (Yao et al. 2021).

Another study was conducted to examine the impact and significance of FT on the level of hypoxia‐inducible factor‐1α (HIF‐1α) and vascular endothelial growth factor (VEGF) expression in cervical cancer tissue of mice. Balb/c nude mice were used as animal models for studying cervical cancer. The animals were injected with HeLa cells to develop the malignancy. The mice were randomly allocated into three groups: the positive control (*n* = 10), the cisplatin group (*n* = 15), and the FT group (*n* = 15). After 31 days of treatment, all mice were euthanized, and tumors were removed and weighed in order to measure the tumor inhibition rate. Concurrently, samples of their malignant cells were collected. The expression levels of mRNA of HIF‐1α and VEGF were measured using RT‐qPCR, while the protein expression level was assessed using western blotting. The mice in the FT group exhibited optimal health and did not manifest any evident negative responses over the course of the pharmaceutical intervention. In contrast, mice present in the cisplatin group had fewer activities, a depressed mood, and reduced appetite. Tumor masses were reduced in mice present in the FT and cisplatin groups compared to the positive control group. The cisplatin group indicated a 56.24% tumor suppression rate in mice, whereas the FT group presented a rate of 50.17%. Protein expression and mRNA levels of HIF‐1α and VEGF showed remarkable inhibition in the mouse tissue cervical cancer FT and cisplatin groups compared to the positive control group. FT has the capacity to hinder the growth of a tumor, reduce levels of HIF‐1α and VEGF mRNA, and protein expression in mouse cervical cancer. FT exhibits inhibitory activity against cervical cancer tumors (Zhang, Chen, et al. 2019; Zhang, Lv, et al. 2019; Zhang, Wang, et al. 2019).

Scientists have taken into consideration a new form of chitosan nanoparticle that has been coupled with folic acid. This nanoparticle aimed to deliver the bioflavonoid genistein to cervical cancer cells, which would undergo selective targeting. Chitosan nanoparticles in their gen‐loaded form GCN and the form conjugated with folic acid FGCN showed a controlled drug release pattern and a decreased size. FGCN showed higher internalization in HeLa cells than GCN. Particular internalization of the FGCN was mostly due to the high affinity of folic acid (FA) for FRs‐α, which are found in large quantities in HeLa cells. These findings demonstrated that FGCN exhibits a distinct attraction for HeLa cells, hence increasing the effectiveness of the therapy. Folic acid‐tagged nano‐formulations had a more cytotoxic impact compared to the nontargeted formulations. Following a 24‐h incubation period, the IC50 value of GEN consistently lowered under treatment with FGCN. The apoptosis research revealed that FGCN nanoparticles exhibited either GCN or the free GEN in relation to their anticancer efficacy (Cai et al. 2017).

### 2.8 Gastric Cancer

Gastric cancer is a highly serious disease on a worldwide scale. Gastric cancer is the sixth most‐identified malignant tumor globally, with around 1 million cases reported annually. This cancer, which ranks third in cancerous fatalities, has a significant mortality rate because it is frequently diagnosed at later stages. The prevalence of stomach cancer is twice as high in males as it is in females. Gastric adenocarcinomas, which constitute the majority of gastric malignancies, display considerable diversity in their structure, growth patterns, origin, cell specialization, and molecular causes (Smyth et al. 2020). *H. pylori* infection is the main factor that causes sporadic distal gastric cancer. Various factors, such as bacterial, environmental, and host variables, work together to promote the repair of damage during the prolonged inflammation generated by *H. pylori* infection and the subsequent formation of cancer. Cancer development associated with inflammation can result from alterations in cellular proliferation, programmed cell death, and particular genetic abnormalities that impede tumor suppression (Van Cutsem et al. 2016). Isoflavones, chemicals included in several plant‐based foods, including peanuts, chickpeas, alfalfa, and mostly soybeans, possess the potential to prevent stomach cancer. Genistein, FT, biochanin A, and daidzein are among the several isoflavones. Genistein is a predominant isoflavone, but daidzein is converted into equol in the colon, which is a more potent precursor in terms of biological activity compared to other isoflavones. Dietary isoflavones have been demonstrated to possess anticancer potential in some types of malignancies due to their capacity to modulate numerous metabolic pathways. Initially, it was hypothesized that the estrogenic characteristics of the soy isoflavones were accountable for their influence on hormone‐dependent diseases, such as prostate cancer and breast cancer. The anticancer activities of these compounds can also be ascribed to their antioxidant and anti‐inflammatory effects (Golpour et al. 2015).

The vitality of gastric cancer cell lines MGC‐803 and SGC‐7901 was reduced by FT, indicating cytotoxic action. In addition, FT inhibited the migration and invasion of GC cells. Additionally, following the findings in vitro, a xenograft model was employed to confirm the efficacy of FT's anticancer impact in vivo. Research has shown that miR‐542‐5p, or microRNA‐542‐5p, is increased in stomach cancer. It did seem pertinent, nevertheless, that FT's anticancer efficacy is miR‐542‐5p‐dependent in GC cells. Both in vitro and in vivo experiments showed that FT significantly reduced GC cell aggressiveness and proliferation (Wang and Zhao 2021).

In order to determine the anticancer effects of genistein on the AGS gastric cancer cell line, which were mediated by the activation of caspase‐3, more study was carried out. The half‐maximal inhibitor concentration and the ability of the AGS gastric cancer cell line were also assessed after 12, 24, and 48 h of treatment with various genistein dosages. Genistein's impact was observed on AGS cells' migratory potential in a wound healing experiment. The effect of genistein on AGS gastric cells' ability to undergo cell death was evaluated through the use of flow cytometry. Colorimetric assays and reverse transcription‐quantitative polymerase chain reactions assessed Caspase‐3 gene expression and enzyme activity, respectively. The findings demonstrated that the IC50 value for genistein was 70 μM after incubation for 24 h. Genistein significantly inhibited AGS cell migration compared to control cells that were not treated. There was a significant increase in caspase‐3 gene expression and induction of early and late apoptosis in the cells treated with genistein. It was observed that caspase‐3 activity has not been enhanced. Genistein moderately inhibited cell death, cellular migration, and CASP3 gene expression in AGS cells, making it an effective anticancer agent (Ghasemkhani et al. 2022).

**FIGURE 3:**
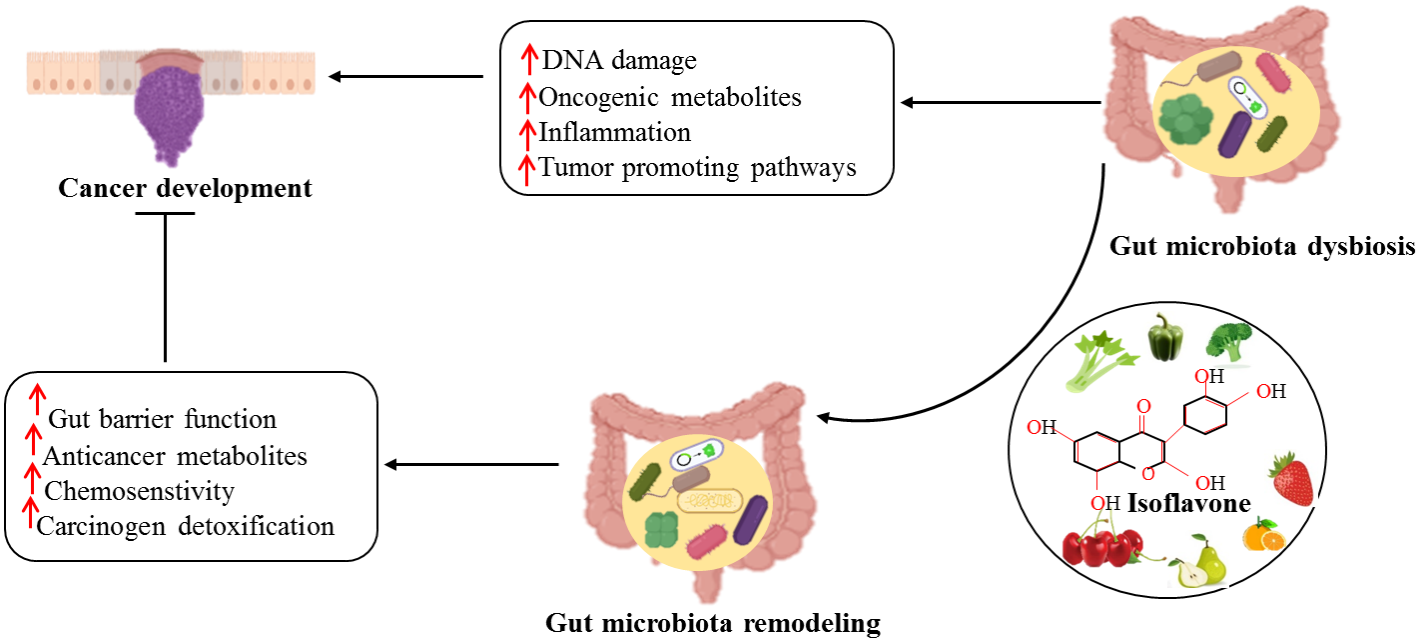
Mechanism of isoflavones to prevent various cancers through gut microbiota.

The researchers investigated the inhibited impact of genistein on cell proliferation, and it was observed that it is associated with the cessation of the G2/M cell cycle and the downregulation of cdc2 activities. This study examines the role of PTEN in regulating genistein‐induced cell cycle arrest at the G2/M phase in SGC‐7901 and BGC‐823 cell lines of gastric cancer. Following a 24‐h treatment, a concentration‐dependent increase in the population of cells in the G2/M phase of the cell cycle was induced by genistein. Genistein induces a prolonged arrest in the G2/M phase of the cell cycle in BGC‐823 and SGC‐7901. This interruption is linked to elevated levels of phospho‐cdc2 (Tyr15) concentrations and lower levels of cdc2 protein concentrations. The findings showed that the application of genistein caused elevated Wee1 levels and a reduction in phospho‐Wee1 (Ser642). Genistein was found to downregulate the phosphorylation of Akt at Thr308 and Ser473 successfully while upregulating the expression of PTEN. The downregulation of PTEN by siRNA reduced cell cycle arrest at the G2/M phase in the presence of genistein. A decrease in phospho‐Cdc2 at Tyr15 was followed by an increase in phospho‐Wee1 at Ser642. Thus, the upregulation of PTEN expression contributed to the induction of G2/M cell cycle arrest by genistein (Liu et al. 2013). Three different gastric cancer cell types, SGC7901, MKN45, and MGC803, were tested to see if FT might suppress their proliferation. From the naturally occurring molecule FT, compound 10 was synthesized using a molecular hybridization method. In addition to an IC50 value of 1.07 μM, the results showed that compound 10 considerably slowed the development of SGC7901 cells. Significantly influencing the Wnt/β‐Catenin and AKT/motor pathways, Derivative 10 can reduce the proliferation and motility of gastric cancer cells from the SGC7901 line. A small number of in vivo investigations have shown that SGC7901 xenograft tumors may be successfully inhibited in live organisms without causing significant weight loss (Yao et al. 2019). Figure 3 illustrates the mechanism of Isoflavone on cancer prevention through gut microbiota.

### 2.9 Kidney Cancer

Renal Cancer is a disorder that is pervasive only in the epithelial lining of renal tubules. This type accounts for about 85% of all cases of invasive kidney carcinoma in adults, making it the most common form. Renal cancer, at an advanced stage, is able to produce a variety of symptoms, such as weight loss, increased body temperature, and greater blood pressure. Even though this is a rare case, renal cancer is still included in the top 10 kinds of cancers; it usually appears after the age of 45. In particular, according to studies, this particular kind of cancer has been proven to appear more in men compared to women and mainly at the age of 60, approximately. Its rates have been continuously increasing over the last decades at an annual rate of 2%–4%. As stated by the very latest cancer statistics, in 2017, more than 64,000 new cases of renal cancer were found in the United States. Additionally, roughly 14,400 persons are estimated to die as a result of renal cancer in the same year (Haque et al. 2017). Phytoestrogens are often used to describe bioactive substances, such as isoflavones (daidzein, genistein, and glycitein), that possess modest estrogenic effects. Isoflavones were hailed as a remarkable natural compounds with the potential to prevent certain serious health problems that were already widespread. Soy product consumption has been linked to a reduction in the occurrence and intensity of chronic illnesses, such as cardiovascular disease, breast cancer, and prostate cancer (Zaheer and Humayoun Akhtar 2017).

Genistein is a kind of polyphenolic isoflavone that is found in soybeans, lupin, fava beans, and new plants like Sohphlang and the large leaf flemingia. It has revealed promising anticancer characteristics in several human cancerous cell lines and tumor models of animals. Genistein was assessed against many kidney cancerous cell lines, including SMKT‐R1, SMKT‐R4, SMKT‐R2, and SMKT‐R3. Genistein treatment suppressed cell development and triggered programmed death of cells (apoptosis) in the RCC cell lines. The duration and application of treatment influenced the effectiveness of this therapy (Bajalia et al. 2022).

Scientists investigated the influence of genistein on the activities of the HOTAIR chromatin remodeling. Cell proliferation and migration in the human renal cell carcinoma cell lines were assayed by the trans‐well migration assay and by the MTS assays in the presence of genistein. Chromatin immunoprecipitation assays were performed to determine the interaction of PRC2 across the promoter region of ZO‐1. Further, activities of ZO‐1 and SNAIL were evaluated using Western blots. The RNA immunoprecipitation methodology compared the interaction occurring between SMARCB1 or ARID1A, PRC2, and HOTAIR. Furthermore, transfection experiments overexpressed EED and HOTAIR while silencing the expression of SMARCB1. Such results concluded that Genistein inhibited human renal cell carcinoma cell lines. Chromatin immunoprecipitation assays revealed that genistein reduced the association of the ZO‐1 promoter with PRC2, resulting in enhanced ZO‐1 expression. RIP experiments showed that genistein is able to significantly inhibit the association between PRC_2_ and HOTAIR and downregulate the development of tumors. Immunoprecipitation further confirmed the downregulation of EED levels in PRC2 by genistein. These results indicate that the lower levels of EED hamper interactions between PRC2 and HOTAIR since genistein leads to an upregulation of the EED expression level, thereby restoring the interactions between PRC2 and HOTAIR, which eventually reduces the ZO‐1 transcription level. Together, the findings depict that genistein suppresses the HCs activity through the upregulation in the ZO‐1 expression since HOTAIR and its activities are inhibited. Further, the RIP assays with HOTAIR demonstrated the mediation of interaction between ARID1A and SMARCB1, both constituents of the human SWI/SNF chromatin complex. The transcriptional process of SNAIL was reduced by genistein via affecting the HOTAIR/SMARCB1 pathway. This was demonstrated by in vitro studies where HOTAIR was overexpressed and SMARCB1 was suppressed while genistein was present (Imai‐Sumida et al. 2020).

Scientists have conducted studies wherein molecular procedures of the CDKN_2_a hypomethylation are associated with antitumor properties in kidney cancer that genistein exudes. The degree of expression of the gene CDKN_2_a was determined by means of a quantitative reverse transcription polymerase chain reaction. The degree of methylation of CDKN_2_a was investigated using methylation‐specific PCR. The analysis of flow cytometry assayed the occurrence of apoptosis. The MTT assay demonstrated cell viability. Results showed that genistein resulted in apoptosis and inhibited the growth of renal carcinoma cells. Besides that, genistein increased the expression of CDKN_2_a while demethylating it. The results further showed that the antitumor effects of genistein may bring about enhanced expression of CDKN_2_a, subsequent to its demethylation, initiating apoptosis or inhibiting the proliferation of renal cancerous cells (Ji et al. 2020).

### 2.10 Lung Cancer

Lung carcinoma is the most widespread form of cancer globally and the leading reason for cancer‐related deaths. It has transitioned from being a rare and mysterious illness. In 2012, the most recent global statistics study of the current age indicated that there were 1.8 million newly identified instances of a certain condition globally, resulting in 1.6 million deaths in the same year. It is a rise from 1.6 million newly identified cases and 1.4 million deaths caused by lung cancer in 2008. Tobacco cigarette usage is the main risk factor for a lung cancer epidemic (De Groot et al. 2018).

The meta‐analysis study resulted in 330,826 candidates being selected for the 23 studies, and the effect of soy and soy products significantly reduced death rates due to cancers. The study resulted in a higher rate of soy consumption being linked with the reduction of mortality risks from lung, colorectal, and gastric cancers. The candidates consuming the highest concentrations of dietary isoflavones depicted a 10% lowered risk of mortality in comparison to the candidate consuming the lowest concentrations. The study showed that the 10 mg/day consumption of isoflavones reduce 9% and 7% of death risks from breast cancer and all other types of cancer, respectively (Nachvak et al. 2019).

These cancers have a malicious and vengeful clinical progression. Soy products have been integrated into the dietary practices of many Asian and Western nations. These meals offer a wide range of nutrients and have a suppressive effect on human ailments. Isoflavones, which include estrogenic and antioxidant effects, are the main bioactive constituents found in soybeans, with a concentration of around 1.5 mg/g. These chemicals could provide advantageous effects on health. Genistein is a prominent isoflavone generated from soybeans that possesses great health advantages. The presence of anticancer properties has been observed in human cancers, specifically hepatocellular carcinoma and head and neck squamous cell carcinoma. The potential antitumor effects of genistein are linked to the noncoding RNAs (Yu et al. 2021). The isoflavones found in *Cicer arietinum* L. are of interest. In addition, biochanin A inhibits the growth of lung cancerous cells and suppresses cell growth by inducing apoptosis and G1/S phase cell cycle arrest. Researchers explored the ability of an herbal ingredient called daidzein‐rich isoflavones aglycone (DRIA) to inhibit growth and the NF‐κB signaling pathway of lung cancer. The study's findings showed that DRIA therapy, in a dose‐dependent manner, suppressed the growth and multiplication of lung cancer cells. The utilization of MTT and colony formation tests corroborated this. In the enzyme‐linked immunosorbent assay, DRIA was seen to reduce levels of interleukin‐8 (IL‐8) and interleukin‐6 (IL6). DRIA decreased elevated levels of p65‐NFκB expression and activation induced by C/EBPβ and TNF‐α treatment. The NF‐κB signaling reduction effect of DRIA was tested at p65‐NFκB protein and mRNA expression levels as well by quantitative real‐time PCR, western blotting, and immunofluorescence labeling. For the enhancement of NF‐κB signaling in the cells, the CCAAT/enhancer C/EBPβ binding protein and exogenous tumor necrosis factor‐α were introduced. The immunohistochemical staining revealed that the expressions of p65‐NFκB and Ki‐67 in A594 tumor xenografts were significantly reduced following DRIA therapy in the mice. This thus suggests that DRIA was able to effectively inhibit NFκB signaling and the growth of lung cancer both in vitro and in vivo (Guo et al. 2020).

Another study was the induction of lung cancer in the previously healthy Swiss albino mice by the polycyclic aromatic hydrocarbon carcinogen, benzo (a) pyrene. Pre‐ and post‐18‐week therapy with prunetin was done for the rats. It was revealed that treatment with prunetin of the lung cancer‐bearing rats promoted a rise in the hematological and immunological indices. The study dealt with the advancement of lung carcinoma that was significantly affected by the oxidation destruction of leukocytes. The therapeutic efficacy of prunetin was evaluated by determining the activity of the metabolizing enzymes in carcinogen‐induced cancer rats. The possible anticancer activities of prunetin were sent for analysis by estimating the levels of anti‐inflammatory markers and the tumor marker CEA. This was further proved through histological examination of the lung tissue. From the results, prunetin greatly enhanced the phagocytic activity, white blood cell count, and activity index in rats that were induced with lung cancer. Furthermore, it decreased levels of oxidative stress in the white blood cells and immunoglobulins. Similarly, the reduction of proinflammatory markers, metabolizing enzymes, and the tumor marker CEA in mice with cancer was also proof that prunetin would possibly be effective in fighting against cancer, the confirmation being again obtained from histological investigation of lung tissue (Yang et al. 2022).

The research was on the role and mechanism of genistein in combinatorial therapy for nonsmall‐cell lung cancer. Genistein is the most common isoflavone compound in soybeans. Genistein has been reported to inhibit the growth of nonsmall‐cell lung cancer (NSCLC). The non‐small‐cell lung cancer cells of the H292 and A549 were treated with genistein. The levels of miR‐873‐5p, hsa_circ_0031250, and FOXM1 associated with circRNA were detected by Western blot analysis. The effects of miR‐873‐5p, genistein, circ_0031250, and FOXM1 on the advancement of nonsmall‐cell lung cancer were assessed via several experimental techniques, including Cell Counting Kit‐8, xenograft model, colony formation, Western blotting, trans‐well assay, flow cytometry, and wound healing. The target connection was investigated using RNA immunoprecipitation and dual‐luciferase reporter analysis. The results showed that genistein, both in dose‐dependent and time‐dependent perspectives, reduced the survival of NSCLC cells. Both nonsmall‐cell lung cancer cell lines and tissues showed a higher expression level of circ‐00312. However, treatment with genistein produced a decrease in its quantity. Downregulation of circ‐0031250 enhanced the inhibitory impact of genistein on cell proliferation, migration, and invasion, thus increasing apoptosis. Nonsmall‐cell lung cancer tissues and cells showed lower levels of miR‐873‐5p. Genistein damaged the influence of circ‐0031250 inhibition on the development of NSCLC, which was attained by circ‐0031250 targeting in reduction of miR‐873‐5p. FOXM1 was expressed under control by the Circ‐0031250/miR‐873‐5p axis. By miR‐873‐5p, this treatment of cells produced control of FOXM1, thus limiting invasion, cell proliferation, and migration and encouraging death. The xenograft model showed that reduction of circ_0031250 improved the inhibitory effect of genistein on the growth of NSCLC cells (Yu et al. 2021).

### 2.11 Oral Cancer

Oral cancer is a type of tumor that forms in the mouth or on the lips. Within the dental domain, 90% of cancerous growths originate from squamous cells, leading to its conventional classification as oral squamous cell carcinoma (OSCC). It has a proclivity for the spread of cancer cells to lymph nodes and shows variable levels of cellular specialization (Rivera 2015). Oral cancer ranks as the 16th most widespread form of cancer and the 15th leading cause of death worldwide. The global incidence of oral cancer (adjusted for age) is four cases per 100,000 persons, with substantial geographical disparities. These disparities are impacted by factors such as gender, age, nation, race, ethnicity, and socioeconomic situations. Oral cavity cancer can be caused by several physical circumstances, environmental variables, and genetic factors that are known to be potential dangers. As a result, an increasing number of cases of oropharyngeal cancers in young people in North America and Europe are caused by “high‐risk” human papillomavirus (HPV) infections (Inchingolo et al. 2020). Soybeans are a rich natural source of isoflavones. Isoflavones belong to the phenolic family of the heterocyclic plants called flavonoids. Three groups of soy isoflavones are glycitein, daidzein, and genistein. Soy isoflavones have several positive benefits, including reducing cholesterol levels, enhancing lipid utilization and digestive system function, and preventing breast, colon, prostate, and oral cancer. Genistein is acknowledged as a naturally occurring compound present in soy that has anticancer properties. Genistein inhibits the growth of malignant cells by S/G_2_‐M cell cycle arrest. It also enhances the apoptotic death of cells in HN4 squamous cell carcinoma of head and neck (HNSCC) cell lines (Valizadeh et al. 2021).

The investigators intended to examine the cancer‐inhibiting impacts of genistein in the development of oral cancer caused by 7,12‐dimethylbenz[a]anthracene (DMBA) on the buccal pouch of the hamsters employing flow cytometry analysis (FMA). Adult male Syrian hamsters were subjected to three different treatments: painting the oral mucosa with paraffin oil only (group 1), mixing DMBA with mineral oil and painting the oral mucosa (group 2), or orally delivering genistein together with DMBA painting (group 2B). Buccal mucosa was used for histological investigation and flow cytometric analysis. These findings suggested that the development of cancer caused by DMBA began in the ninth week. Progressive indications became evident in the following weeks, reaching a peak with the formation of significant ulcerative oral masses with outgrowth nodules in the 21st week. The histological investigation revealed that the well‐differentiated oral squamous cell carcinoma (OSCC) exhibited malignant characteristics in the underlying tissues starting from the 12th week. The genistein‐induced clinicopathological abnormalities were characterized by mild epithelial dysplastic alterations, which manifested 6 weeks after the onset of changes observed in the DMBA‐painted animals. During the previous 6 weeks, there has been a modest progression with the absence of dysplastic alterations. Flow cytometry analysis revealed that the DMBA exposure led to a substantial rise in S‐phase fragment (SPF) value and a notable alteration in DNA propagation activity. Additionally, aneuploid DNA patterns were seen in 47.22% of the hamsters. After genistein therapy, the levels of the S‐phase fragment (SPF) fell significantly (Hussein et al. 2022). Scientists explored the epigenetic regulatory mechanism of the genistein nanoformulation (GLNPs) to assess its effectiveness in selectively triggering apoptosis in OSCC. The genistein nanoformulation was synthesized utilizing lactalbumin. The process of epigenetic alteration and apoptosis mediated by the genistein‐loaded nanoparticles was examined in the JHU011 cell line of OSCC and the L929 cell line of fibroblast using ChIP‐qPCR assay, immunofluorescence assay, and Western blotting assay. The findings demonstrated that the administration of GLNPs specifically induced programmed cell death in OSCC, while having no effect on normal fibroblast cells. The careful impact in OSCC is attained by a rise in the reactive oxygen species (ROS) formation, which leads to the movement of Bax to mitochondria and activation of caspase 3. In addition, GLNPs caused the cessation of epigenetic transcription repression by simultaneously reducing the expression of polycomb group proteins (PcG) EZH2 and Bmi 1, and their downstream substrates, H3K27me3 and UbH2AK119. The results have important therapeutic suggestions for the treatment of oral squamous cell carcinoma (OSCC). Ultimately, it was concluded that GLNPs control the activity of EZH2 by suppressing 3PK and promoting proteasomal‐mediated degradation. The physical association between the 3PK protein and the EZH2 protein, as well as its supporter region (−1107 to −1002), has been identified. This event indicates that the 3PK could have a notable influence on the epigenetic control of OSCC and the expression of EZH2. In addition, the preparation exhibited better compatibility, ability to disperse in water, and greater distribution within the body when tested in living organisms. The data presented provided proof that the GLNPs have the capacity for selective apoptosis and alleviate epigenetic transcriptional suppression in human oral squamous cell cancer (Dev et al. 2021). Scientists investigate cellular signaling routes and the anticancer properties of biochanin‐A in FaDu pharyngeal squamous cancer cells. Biochanin‐A caused cell death in FaDu cells by enhancing their cytotoxicity in a way that was dependent on the dose and duration of exposure. The FaDu cells were exposed to biochanin‐A for a duration of 24 h, leading to an augmentation in the quantity of the cells exhibiting nucleus aggregation and the population undergoing apoptosis. In addition, FaDu cells that were treated with biochanin‐A showed a dose‐dependent increase in extrinsic apoptotic factors, including caspase‐8 and FasL, which are their downstream targets. Furthermore, biochanin‐A reduced the presence of natural anticell death factors, including Bcl‐xL and Bcl‐2, while enhancing the quantity and activation of natural cell death factors, including caspase‐9 and Bad (Cho et al. 2017).

### 2.12 Pancreatic Cancer

Pancreatic ductal adenocarcinoma (PDAC) is a very rare form of cancer, with a predicted 60,430 cases expected to be identified in the United States in 2021. PDAC is anticipated to become the second most common reason for cancer‐related deaths through 2030, with an annual growth rate of 0.5%–1.0%. In 2012, pancreatic cancer ranked as the 11th most prevalent form of cancer globally, impacting almost 338,000 people (Park et al. 2021). Developed countries have the highest rates of both death and occurrence of pancreatic cancers. Tobacco usage is a well‐established factor in the development of pancreatic cancer. The 5‐year existence rate is around 6% (ranging from 2% to 9%), but the disparity between established and developing nations is insignificant. Adenocarcinoma, accounting for around 85% of pancreatic cancer occurrences, and pancreatic endocrine tumors, making up fewer than 5% of all cases, are the two main types of pancreatic cancer (Ilic and Ilic 2016). Isoflavones, which are polyphenol chemicals, are often known as phytoestrogens and are used in the therapy of pancreatic cancer. Genistein demonstrates both antioxidant and anticancer characteristics. Biochanin A is an isoflavone found in red soy or clover is known for its anticancer capabilities in several types of cancers (Li et al. 2015). Genistein is a flavonoid that is naturally present in pulses and specific herbal treatments. Genistein is classified as a soy isoflavone and has been found to be effective in preventing hormone‐related illnesses such as chronic renal disease, breast cancer, osteoporosis, and cardiovascular diseases (El‐Kordy and Alshahrani 2015). Puerarin, also known as 7,4′‐dihydroxyisoflavone‐8‐β‐glucopyranoside, is a naturally occurring flavonoid chemical that has been extracted from the conventional Chinese herbs *Radix puerariae*. Puerarin exhibits the capability to impede cellular growth and trigger programmed cell death, hence potentially exerting anticancer properties (Zhu, Lu, et al. 2021; Zhu, Xiao, et al. 2021).

The research has employed both in vivo and in vitro testing to assess the tumor‐repressive impacts of puerarin. The study examined the influences of puerarin on proliferation, programmed cell death, motion, and invasion of PCCs, as well as tumor growth and metastasis in a mouse model of PDAC xenografts. Fundamentally, results demonstrated significant inhibition of PCC growth by puerarin therapy. Puerarin caused PCCs to lose their balance between Bcl‐2/Bax and subsequently triggered mitochondrial‐dependent apoptosis. PCC invasion and migration were also suppressed by puerarin through counteracting EMT. Administration of puerarin suppressed PDAC development in a nonfunctional immunocompromised mouse model. It was proposed that the therapeutic effectiveness of puerarin on PDAC is accomplished by blocking the Akt/mTOR signaling pathway. This is due to the fact that puerarin binds to the mTOR protein's kinase, which in turn affects the function of the amino acid deposits involved in binding the ATP‐Mg2+ complex. An additional study found that activating mTOR reversed puerarin's inhibitory effects on PCCs, indicating that mTOR may play a significant mediating role in puerarin's antitumor activities in PDAC. Additionally, puerarin slowed metabolism and glucose absorption by reducing the rates of oxygen consumption and extracellular acidification, both of which were dependent on the glucose transporter GLUT1 and HIF‐1α (Zhu, Lu, et al. 2021; Zhu, Xiao, et al. 2021).

### 2.13 Prostate Cancer

Prostate cancer ranks as the fifth leading cause of mortality worldwide and is the second most prevalent disease in males. Prostate cancer can show no symptoms in the early stages and often has a slow and nonaggressive progression, which means that vigilant monitoring is sufficient. In 2018, there were 1,276,106 new cases of prostate cancer worldwide, with a greater occurrence in industrialized nations. American and African men have higher occurrence rates and more severe manifestations of the prostate tumor compared to White males (Rawla 2019). Isoflavone is a kind of phytoestrogen with a moderate estrogenic impact and has molecular structures that are comparable to those of animal estrogen. Biochanin A, Genistin, equol, glycitin, and daidzin are the main isoflavones (Zhang et al. 2016). These are di‐phenolic chemicals specifically engineered to bind to estrogen receptors and provide modest estrogen‐like actions. Therefore, these are considered phytoestrogens. Genistein and daidzein have also exerted anticarcinogenic activity, and it is not necessarily linked to hormonal activity. In vitro, genistein has been shown to affect many cellular mechanisms like tumor metastasis, cell proliferation, tumor cell invasion, apoptosis, cell cycle regulation, and angiogenesis. In addition, it has been shown to prevent the secretion of PSA in the androgen‐sensitive human prostate adenocarcinoma (LNCaP) cells (Van Die et al. 2014).

The scientists examined the effects of genistein on major cell signaling pathways in PC3 prostate cancerous cells, including caspase‐3 and p38MAPK. Next, the amounts of p38MAPK and caspase‐3 protein in the cells and gene expression were measured. The gelatinase activity of matrix metalloproteinase‐2 and enzyme activity of caspase‐3 were measured, while the proliferation and migration potentials of the PC3 cell line were evaluated. These results indicated that the genistein‐induced apoptosis was mediated through the enhancement of the enzymatic activity of caspase‐3, intracellular protein levels, and gene expression. Genistein strongly suppressed the metastatic potential of PC3 cells by inhibiting MMP2 action and downregulating both p38MAPK protein levels and gene expression, which in turn retarded cell multiplication. It is concluded that genistein has positive anticancer properties on PC3 cells through the metastatic reduction potential and modulation of p38MAPK and caspase‐3 pathways at different protein and transcriptional levels in PC3 cells (Shafiee et al. 2022).

Scientists conducted a research on the PC3, LnCaP, and DU145 human prostate tumor cell lines to examine the inhibitory impacts of S‐equol. S‐equol is an isoflavandiol, and it is produced from daidzein by bacteria in the intestines. Results demonstrated that both R‐equol and S‐equol suppressed the development of the three cell lines. Another study discovered that S‐equol triggered apoptosis by increasing the expression of Fas ligand (FasL) and proapoptotic bim ligand. It also halted G2/M phase cell cycle arrest in the PC3 cells by reducing the levels of CDK1 and Cyclin B1, while promoting the activity of CDK inhibitors p27 and p21. In addition, S‐equol increased the level of FOXO_3_a expression, reduced the level of p‐FOXO_3_a expression, and enhanced the nuclear firmness of FOXO_3_a. The expression of MDM2 is decreased by S‐equol, which acts as an E3 ubiquitin ligase for p‐FOXO_3_a. As a result, the proteasome was incapable of breaking down p‐FOXO_3_a. The mechanistic research of S‐equol revealed its ability to target the Akt/FOXO_3_a pathway, which plays a critical part in the existence of prostate cancer cells, the progression of the cell cycle, and apoptosis. In addition, the growth of the PC_3_ xenograft tumor in the BALB/c nude mice was suppressed by S‐equol therapy. The research's findings suggest that S‐equol has significant antiprostate tumor properties both in laboratory conditions (in vitro) and in living organisms (in vivo). The study also indicates that the anticancer benefits of S‐equol are likely attributed to its ability to activate FOXO_3_a through a particular route involving Akt as well as its ability to suppress the expression of MDM2 (Lu et al. 2016).

**FIGURE 4:**
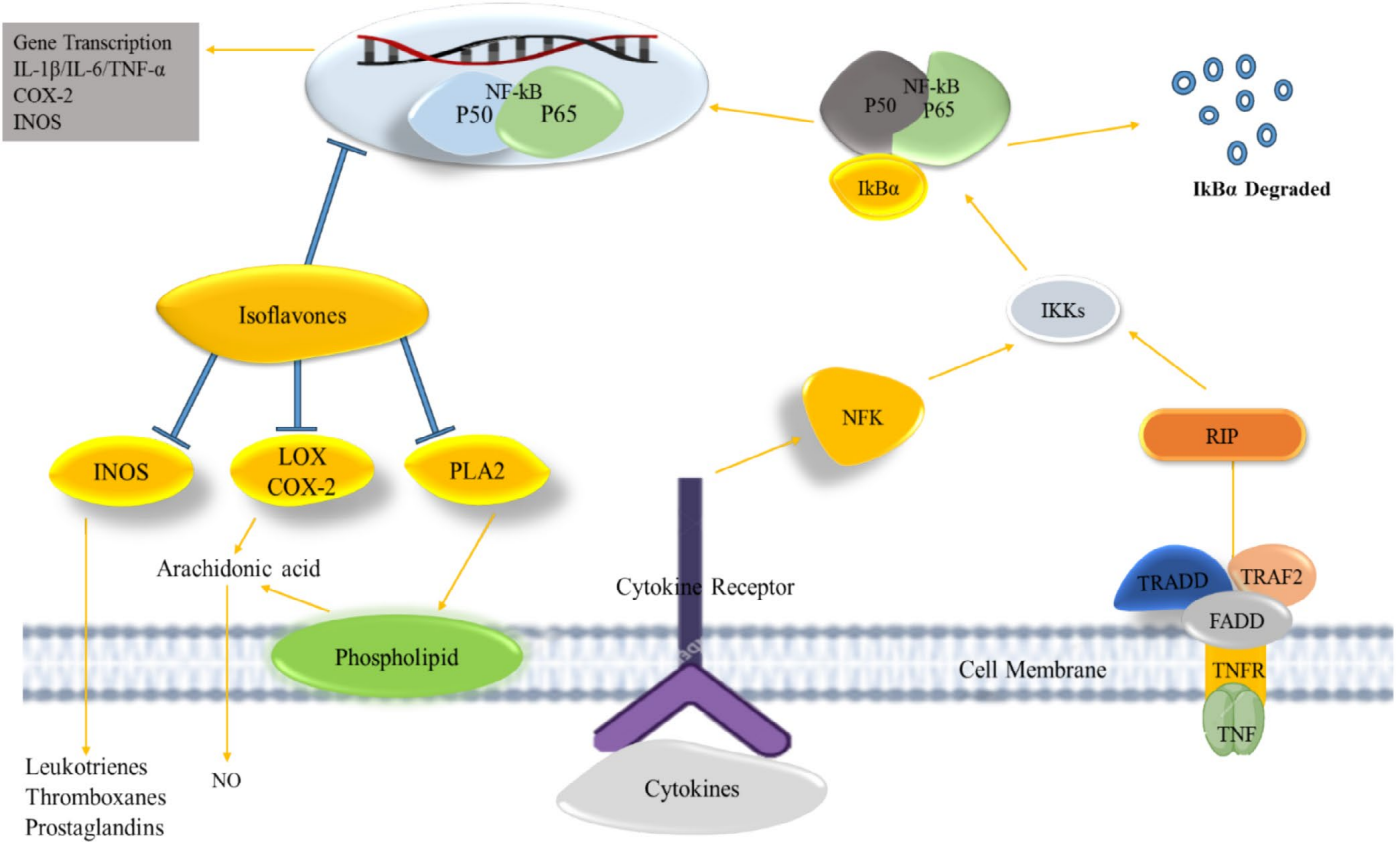
Mechanisms of isoflavones to treat the various hormonal cancers.

In another investigation, scientists analyzed the degree to which FT hinders epithelial–mesenchymal transition of PCa cell lines that are hormone resistant. The CCK8 assays and RTCA system were used to assess the migration, invasion, and propagation of PCa cells. Immunoblotting methods detected the expressions of the MAPK pathway and EMT‐related markers, quantitative reverse transcription polymerase chain reaction, and an immunofluorescence labeling method. Gene expression profiling in human prostate cancer tissue was probed utilizing EGR1 expression techniques and RNA‐sequence analysis. These results indicated that FT had a powerful inhibitory effect on the growth, movement, and infiltration of the PCa cells. It also upregulates the expression of the protein E‐cadherin and downregulates the levels of the fibronectin protein and phosphorylated JNK and ERK1/2. Furthermore, it reduced the mRNA quantities of snail, fibronectin, and slug. It has been demonstrated that FT exerts a strong effect on increasing EGR1 by western blotting, bioinformatics analysis, and RT‐qPCR. Moreover, the expression level of EGR1 was lower in human prostate cancer tissue than in controls and still lower in higher Gleason grade prostate cancer than in lower Gleason grade prostate cancer. FT may enhance the epithelial–mesenchymal transition (EMT) in hormone‐resistant prostate cancer (PCa) cells, which in turn reduces tumor aggressiveness. Possible processes involve a consequent increase in EGR1 expression and deactivation of MAPK signaling (Liang et al. 2022). Figure 4 shows the anticancer mechanism of isoflavones.

### 2.14 Skin Cancer

Skin cancer is the most prevalent form of cancer, encompassing both nonmelanoma skin cancer (NMSC) and MM. MM in those aged 50 and over is increasing at a rate of 0.6% per year, but nonmelanoma skin cancer cases are also on the rise. At 4.5% of all new tumor cases, over 76,380 fresh cases of cutaneous melanoma were found in 2016 (Apalla et al. 2017). The skin is the primary defense mechanism of the human body against external threats. The skin consists of three separate layers: hypodermis, epidermis, and dermis. Neoplastic development and extension in the epidermis may be categorized into three distinct phases: start, promotion, and proliferation of tumor cells. Starting can be triggered by external agents like poisons, viruses, and ultraviolet radiation or by endogenous causes like swelling. The irreversible harm to the skin cells induced by certain factors becomes apparent when cells are not able to restore their regular function and advance to neoplastic development. Squamous cell carcinoma and basal cell carcinoma make up the bulk of nonmelanoma skin malignancies (NMSCs) (Islam, Islam, et al. 2020). The advantageous impacts of phytochemicals on several illnesses, including cutaneous cancer, are generally acknowledged. Isoflavones have demonstrated cancer‐chemopreventive characteristics in several types of malignancies, including neuroblastoma, nonmelanoma and melanoma cutaneous cancers, and breast cancer. Genistein has been shown to have anti‐angiogenic characteristics, cause cell cycle arrest, enhance cell death, and decrease cancer growth and spread. The use of isoflavone provided protection against both photoaging and UV‐induced skin cancer in people. This protection was achieved by lowering the occurrence of sunburn caused by UV exposure (Ng et al. 2018).

Researchers have found that scandenolone is a naturally present isoflavonoid derivative found in *Cudrania tricuspidata* fruit, has an antineoplastic effect in the SK‐MEL‐28 cells, specifically via triggering apoptosis and inhibiting autophagy flow. Moreover, scandenolone suppressed cell migration, causing cell cycle arrest at the G2/M phase. Scandenolone was shown to directly connect to the ATP‐binding site of EGFR and active kinase during the investigation of intracellular molecular targets. As a result, it downregulates the AKT/mTOR/ERK signaling cascade. These findings indicate that the scandenolone derived from the *Cudrania tricuspidata* fruit effectively enhanced the susceptibility of the SK‐MEL‐28 cells to programmed cell death and inhibited growth, demonstrating a notable promise for the management of melanoma (He et al. 2017).

Anticancer activity against human malignant melanoma cells of SK‐Mel‐28 was investigated for Biochanin A. The cytotoxic activity of Biochanin A was tested using the MTT assay. The effect on cellular invasion and motility was tested by the in vitro wound repair and invasion experiments. The influence on cell shape and programmed cell death was studied by fluorescence microscopy with acridine orange/propidium iodide. The expression of mitogen‐activated protein kinase (MAPK) and nuclear factor (NF)‐κB proteins was measured with Western blotting. These results showed that Biochanin A could successfully prevent the proliferation of the SK‐Mel‐28 cell in a manner dependent on both the dose and the duration of treatment. The administration of Biochanin A effectively induces apoptosis of the cells and is directly proportional to the dose. Furthermore, Biochanin A also illustrated a dose‐dependent reduction of cell invasion and migration but, on the other hand, an enhancement of the expression of key proteins involved in the MAPK and NF‐κB signaling pathways (Xiao et al. 2017).

The researchers investigated the anticancer effects of genistein, a phytoestrogen isoflavone, on squamous cancer SK‐MEL‐28 (SCC) cells through invasion, MEK/ERK/JNK signaling, migration, and cellular apoptosis: The effects on programmed cell death were analyzed using the Comet test and DAPI staining, whereas cell growth was examined using the Cell Counting Kit‐8 (CCK‐8) assay. Experiments were conducted using fluorescence microscopy. The trans‐well technique assayed Cell invasion and migration, while the effect on the expression of the MEK/ERK/JNK proteins was streamed through western blotting. These results demonstrated that the isoflavone exerted dose‐dependent cytotoxic effects on the cells, with an IC50 value of 14.5 μM. As far as DNA damage and apoptosis are concerned, further fluorescence microscopic analysis indicated DNA damage induced along with the initiation of apoptosis in a dose‐dependent manner. This isoflavone not only downregulated the expression of matrix metalloproteinase (MMP)‐9 but also downregulated cell invasion and migration in a manner that was dose‐dependent. Isoflavone was capable of dampening the expression of p‐JNK, but without displaying any observable influence on the overall activity of JNK. On the other hand, the levels of the p‐ERK and p‐MEK proteins were also profoundly and dose‐dependently suppressed. Genistein demonstrates significant anticancer efficacy in human SCC cells SK‐MEL‐28 by suppressing the JNK and MEK/ERK signaling pathway, promoting apoptosis, causing damage to DNA, and limiting cell invasion and migration (Li et al. 2020). Table 1 reports the differences in in vitro and in vivo studies of isoflavones against cancer.

**TABLE 1:** Published data on in vitro and in vivo studies of isoflavones against cancer.

Types of cancer	In vitro/in vivo	Cell lines	Source/intervention	Effect/mechanism	References
Breast cancer	In vitro	MCF‐7 cells, SKBR‐3 cells, and HUVEC cells	Isoflavones	Catechin presented stimulatory impact on cell propagation of HUVEC cell line	Lin et al. (2019)
Breast cancer	In vitro	MCF‐7 and MCF‐7 HER2	Isoflavones	Enhanced protein expression	Basu and Maier (2018)
Breast cancer	In vitro	MDA‐MB‐231 and MCF‐7	Isoflavones	Stat3, Akt, and PRAS40 phosphorylation	Zhu et al. (2018)
Prostate cancer	In vitro	PC‐3 and DU‐145	Isoflavones	Suppression of growth factor tyrosine kinase	Van der Eecken et al. (2023)
Prostate cancer	In vitro	VeCaP cell line	Isoflavones	High concentrations of genistein inhibit PSA expression	Sivoňová et al. (2019)
Prostate cancer	In vitro	LNCaP and PC‐3 cell lines	Isoflavones	Decreased cancer cell adhesiveness	Ajdžanovic et al. (2019)
Cervical cancer	In vivo	HeLa cell lines	Isoflavones	Low expression levels of ERα and ERβ	Yamashita et al. (2022)
Cervical cancer	In vitro	HeLa cell lines	Isoflavones	Activation of ER, PI3K/Akt and NF‐κB pathways	Chen et al. (2018)
Hepatic cancer	In vitro	HEPG2/C3A cell lines	Isoflavones	The levels of CYP1A1 and CYP1B1 mRNA were increased, while those of CYP2D6, CYP26A1, and CYP26B1 mRNA were decreased	Lepri et al. (2018)
Hepatic cancer	In Vivo	Hep3B cell lines	Isoflavones	Elevating ROS levels within cells and triggering stress in the endoplasmic reticulum	Zhang, Chen, et al. 2019; Zhang, Lv, et al. 2019; Zhang, Wang, et al. 2019
Colon cancer	In vivo	HT‐29 cell line	Isoflavones	Activation of p53 and Inhibition of Human Topoisomerase II	Schroeter et al. (2019)
Colon cancer	In vitro	NCM460 and SW620 cell lines	Isoflavones	Reduce the apoptosis of NCM460 and inhibiting the proliferation of SW620	Yu et al. (2023)
Colon cancer	In vitro	HT29 and SW620 cell lines	Isoflavones	Reduction in colon cancer cell viability and halt in the G2/M cell cycle	Alorda‐Clara et al. (2022)
Lung cancer	In vivo	A594 and 95D cells	Isoflavones	NF‐κB inactivation in lung cancer and decreased pro‐inflammatory cytokine production	Guo et al. (2020)
Lung cancer	In Vivo	H292 and A549 cell lines	Isoflavones	Suppression of nonsmall‐cell lung cancer (NSCLC) cell viability as well as of cell migration, invasion, and proliferation	Yu et al. (2021))
Gastric cancer	In vivo	GCIY and MKN‐1 cell lines	Isoflavones	Enzyme activation of mitochondrial apoptotic caspase 3 and 9 and cell cycle arrest at G 2 phase	Hikita et al. (2021)
Gastric cancer	In vitro	SGC‐7901 cell lines	Isoflavones	Reduced N‐cadherin and Twist 1 expression and increased E‐cadherin expression	Cayetano‐Salazar et al. (2021)
Ovarian cancer	In vivo	ES2 and OV90 cell lines	Isoflavones	Inactivation of ERK1/2 and PI3K/AKT causes G0/G1 cell phase arrest	Park et al. (2018)
Ovarian cancer	In Vivo	SKOV3 cell lines	Isoflavones	Causing cell death and a halt in cell cycle progression while blocking the Raf/MEK/ERK pathway	Hua et al. (2018)
Gall bladder cancer	In vivo	GBC‐SD cell line	Isoflavones	Minimized growth of gallbladder cancer by MCM complex downregulation	Geng et al. (2022)
Gall bladder cancer	In vitro	TJ‐GBC2 cell line	Isoflavones	By focusing on the PTEN/PI3K/AKT signaling pathway, it causes cell death and blocks cell migration and invasion	Huang et al. (2020)
Neuroblastoma	In vitro	SH‐SY5Y cells	Isoflavones	To prevent ATR‐induced neurotoxicity, isoflavones‐induced BEX2‐dependent autophagy	Li, Ma, et al. (2018); Li, Yu, et al. (2018)

## 3 Conclusion

Isoflavones are naturally occurring polyphenols with many characteristics that effectively hinder the proliferation of cancer cells. In the current review, the data summarized depict that the isoflavones have prominent potential in the prevention and treatment of breast, GIT, liver, prostate, and other various cancers. Isoflavones may possibly have therapeutic benefits in the treatment of cancers in other anatomical sites. Isoflavones have shown promising results in many studies by impeding the proliferation and division of cancer cells through various pathways. It has the ability to treat all kinds of cancer cells. Beyond triggering death in MCF‐7 breast cancer cells, isoflavones hindered the functioning of Nrf2 and the regulation of its genes. The mechanism of action of isoflavones involves the generation of caspases and the inhibition of cancer‐causing signaling pathways connected with Akt. Isoflavones activate AMPK, which inhibits Akt, slows down the development of human lung cancer cells, and induces apoptosis, or programmed cell death. Isoflavones inhibit the proliferation of ATC cells by inducing cell death. Isoflavones have been shown to accelerate autophagy in EC cells by blocking the Akt/mTOR signaling pathway mediated by reactive oxygen species (ROS), leading to an increase in autophagic degradation. Scientific studies have demonstrated that isoflavones consumption decreases a number of pathologies, including astrocyte edema, liver necrosis, caspase‐3 protein production, and Toll‐4 receptor gene (TLR‐4). Many operations, that is, activation of GPER and reduction of ROCK1, TAGLN2, and FCHO2 expressions, are involved in the prevention of cancer development. In prostate cancer cells, isoflavone administration stimulated mitogen‐activated protein kinases (MAPK), namely ERK1/2 and P38. However, it may reduce levels of AKT, P70S6K, S6, and P90RSK proteins and may impair phosphoinositide 3‐kinase (PI3K) activity. The levels of phosphoinositide 3‐kinase (PI3K) were found to be lower when isoflavones were present. Evidence from in vitro, in vivo, and clinical studies highlights their role in suppressing various cancers, including breast, prostate, lung, gastric, and colon cancer, primarily through the regulation of signaling pathways, that is, MAPK/ERK and PI3K/AKT/mTOR. Despite these promising findings, several challenges remain, including limited bioavailability, metabolic variability, and inconsistencies in clinical outcomes. To translate these findings into clinical practice and dietary recommendations, future research should focus on conducting well‐controlled clinical trials to establish optimal dosage, efficacy, and long‐term safety. Investigating their potential synergistic effects with conventional cancer therapies could provide valuable insights into combination treatment strategies that enhance efficacy while minimizing side effects. While isoflavones present a promising natural alternative for cancer prevention and adjunct therapy, further clinical validation and mechanistic exploration are essential to fully establish their therapeutic potential. Advancing these research directions will aid in developing evidence‐based dietary recommendations and targeted therapeutic interventions for cancer patients and at‐risk populations.

## Author Contributions

**Muhammad Hammad Ul Hassan:** conceptualization (equal), writing – original draft (equal). **Muhammad Shahbaz:** conceptualization (equal), writing – original draft (equal). **Muhammad Imran:** supervision (equal), visualization (equal). **Ushna Momal:** writing – review and editing (equal). **Hammad Naeem:** writing – review and editing (equal). **Ahmed Mujtaba:** resources (equal), software (equal), validation (equal). **Muzzamal Hussain:** writing – original draft (equal). **Muhammad Junaid Anwar:** writing – review and editing (equal). **Suliman A. Alsagaby:** resources (equal), software (equal), validation (equal). **Waleed Al Abdulmonem:** methodology (equal), writing – review and editing (equal). **Tadesse Fenta Yehuala:** supervision (equal), visualization (equal). **Mohamed A. Abdelgawad:** methodology (equal), writing – review and editing (equal). **Ahmed H. El‐Ghorab:** data curation (equal), investigation (equal). **Samy Selim:** methodology (equal), writing – review and editing (equal). **Ehab M. Mostafa:** data curation (equal), investigation (equal).

## Conflicts of Interest

The authors declare no conflicts of interest.

## Data Availability

The data that support the findings of this study are available upon request from the corresponding author.
